# Pan‐cancer atlas of cellular architecture reveals a nearest‐neighbour distance‐associated biomechanical‐immune axis involving CD4+ memory T cells

**DOI:** 10.1002/ctm2.70742

**Published:** 2026-07-21

**Authors:** Linglong Huang, Arne Östman, Yunfan Sun

**Affiliations:** ^1^ Department of Oncology‐Pathology Karolinska Institutet Stockholm Sweden; ^2^ Department of Hepatobiliary Surgery and Liver Transplantation Zhongshan Hospital Fudan University Shanghai China

**Keywords:** CD4 memory T‐cell, digital pathology, Hippo pathway, mechanical stress, multi‐omics integration, nearest neighbour distance, spatial analysis, survival analysis, tumour microenvironment

## Abstract

**Background:**

The systematic link between cellular spatial organization and its biomechanical consequences remains a critical knowledge gap in human oncology.

**Objective:**

To quantitatively map the cellular architecture of solid tumours and elucidate its mechanistic impact on mechanical signalling, immune infiltration, and patient survival.

**Methods:**

We developed a scalable digital pathology framework, processing 7910 H&E whole‐slide images across 21 solid tumours. A deep learning pipeline was employed to segment over 4.7 billion nuclei, enabling the calculation of cell density and nearest neighbour distance (NND) as key spatial metrics. To bridge morphology and function, we integrated these metrics with bulk RNA‐seq data from 19 TCGA cohorts (*n* = 7401) using rigorous histological matching. Furthermore, to resolve microenvironmental heterogeneity, we performed unsupervised clustering of over 60 000 T‐cell transcriptomes from independent cohorts. These findings were validated through high‐resolution Visium‐HD spatial transcriptomics to correlate physical proximity with localized gene expression.

**Results:**

While tumours exhibited significant heterogeneity, NND, but not cell density, emerged as a primary determinant of biomechanical and immune signatures. A trend‐level association was observed between lower NND and higher Hippo/YAP/TAZ pathway activity across nine matched cancer types (Spearman's *ρ* = ‐.65, *p* = .058, *n *= 9). In addition, lower NND was significantly correlated with increased CD4^+^ memory T‐cell (CD4^+^ T_Mem_ cell) abundance (Spearman's *ρ* = ‐.86, *p* < .01). Single‐cell analyses confirmed that CD4^+^ T_Mem_ cells intrinsically express mechanical stress signalling markers, which spatial transcriptomics localized to CD4^+^ T_Mem_ cell aggregation zones characterized by high pathway activity. Clinically, this spatial‐mechanical‐CD4^+^ T_Mem_ cell axis was associated with prognosis in breast, oesophageal, liver, and lung adenocarcinomas, where high mechanical signalling generally predicted poor outcomes but could be modulated by CD4^+^ T_Mem_ infiltration levels.

**Conclusion:**

Our study identifies low NND as a spatial correlate of biomechanical crowding that is associated with CD4^+^ T_Mem_ cell programming and adverse clinical outcomes. By integrating deep learning‐based spatial metrics with multi‐omics, we highlight spatial mechanics as a critical, potentially targetable dimension of the tumour microenvironment for future immunotherapies.

## Highlights

1. NND extracted from routine H&E slides serves as a spatial proxy for mechanical crowding across 21 cancer types.
2. Low NND is associated with Hippo/YAP/TAZ pathway activation and CD4^+^ T_Mem_ cell enrichment, validated by single‐cell and spatial transcriptomics.
3. This spatial‐mechanical‐immune axis carries prognostic significance, with potential implications for spatially informed immunotherapy.

## Graphical abstract

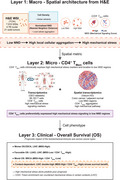

## 1 INTRODUCTION

The spatial architecture of solid tumours, including fundamental features like cell density and how cells are distributed, is more than just a structural trait. It directly reflects the biomechanical state of the tissue. Mechanical stress, caused by cellular crowding and physical compression, maybe vary considerably across tumour types. This variation is thought to influence how tumours progress and how they respond to therapy.^1^, ^2^ While modern oncology has understandably focused on molecularly defined cell types, such as distinguishing cytotoxic T‐cells from regulatory T‐cells,^3^, ^4^, ^5^, ^6^, ^7^, ^8^ this focus has come at the expense of studying broader, pan‐cellular spatial organization and its biomechanical implications. As a result, some fundamental questions remain unanswered. For instance, it remains unclear whether aggressive and expansive tumours like sarcomas naturally experience less mechanical stress than carcinomas.^9^ Similarly, whether a universal spatial‐biomechanical mechanism governs tumour organization across different cancer types is an open question. Characterizing these global architectural traits is fundamental, as they may capture emergent biological properties and clinical outcomes that remain invisible to single‐cell molecular profiling alone.

Routine haematoxylin‐and‐eosin (H&E) stained whole‐slide images are the most abundant and clinically established source of histopathological data worldwide. However, the quantitative spatial information they contain, along with the mechanical stress states that can be inferred from it, remains largely unexplored at scale. We still lack a systematic, pan‐cancer atlas that links features like cellular density and spatial patterning to downstream signalling pathways and immune cell behaviour.

Here, we bridge this gap by introducing a digital‐pathology framework. We used it to quantify tumour cellular architecture to assess the biomechanical context across 21 solid tumour types. Applying deep learning‐based nuclear segmentation to 7910 H&E‐stained whole‐slide images (WSIs), we mapped the spatial coordinates of more than 4.7 billion cells. From this, we derived two core metrics: cell density and nearest‐neighbour distance (NND), with NND serving as a spatial proxy for local mechanical stress.

We then integrated these spatial‐biomechanical features with transcriptomic data. This analysis identified the Mechanical/Hippo/YAP/TAZ pathway as a molecular correlate of high cellular crowding at a trend level. To explore the microenvironmental heterogeneity, we analysed over 60 000 single T‐cell transcriptomes and found this pathway was specifically enriched in CD4^+^ memory T‐cells (CD4^+^ T_Mem_ cells). We confirmed this spatially using Visium‐HD transcriptomics on colon tissues, which directly showed these CD4^+^ T_Mem_ cells preferentially localizing within high‐mechanical‐stress niches. Clinically, this spatial‐mechanical‐immune axis was associated with poorer survival across multiple cancer types, though the strength of association varied by tumour type.

In summary, this study presents a pan‐cancer atlas of tumour cellular architecture and its biomechanical imprint. It establishes NND as a quantifiable, pathology‐informed spatial biomarker. More importantly, it reveals how spatial crowding can co‐occur with mechanical signalling and influence immune cell positioning in human tumours. By connecting macroscopic tissue organization to molecular and clinical phenotypes, this work points towards new therapeutic strategies that target the biomechanical tumour microenvironment (TME).

## 2 RESULTS

### 2.1 A pan‐cancer atlas of cellular architecture across 21 solid tumours

**FIGURE 1:**
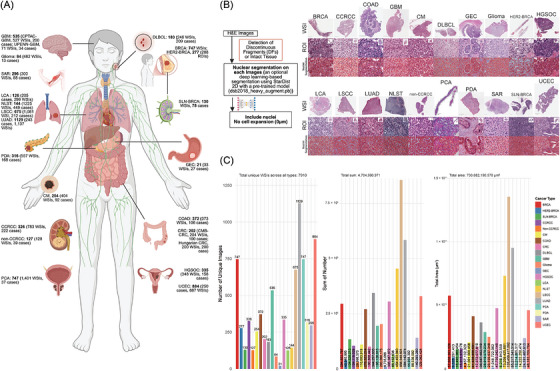
A pan‐cancer analysis of cellular architecture in solid tumours. (A) The diagram illustrated the number of images filtered from The Cancer Imaging Archive (TCIA) for further analysis. The values in bold indicated the final number of whole slide images (WSIs) for each cancer type or dataset. (B) Workflow and representative results of nuclear segmentation in H&E‐stained images. H&E WSIs were processed by a deep learning model to segment cell nuclei. For each cancer type, a representative WSI alongside a zoomed‐in view with overlaid segmentation results was shown. Scale of visualization. All whole‐slide images (WSIs) are presented at a scale bar of 2 mm. The corresponding whole‐slide images (WSIs) and regions of interest (ROIs) for each tumour type are displayed at the following magnifications, with scale bar lengths of 20 and 50 µm, respectively. (Note: ROI scale bars were chosen to best illustrate the characteristic architecture of each tumour type.). (C) Data summary plots. Bar plots are presented as follows: one depicting the number of quality‐controlled images analysed per cancer type; and another set presenting the total count of segmented nuclei and the corresponding total nuclear area (µm^2^) for each cancer type. BRCA: Breast Cancer/Invasive Carcinoma, Invasive Breast Carcinoma; CCRCC: Kidney Cancer/Clear Cell Renal Cell Carcinoma; Non‐CCRCC: Kidney Cancer/Non‐Clear Cell Renal Cell Carcinoma; CM: Cutaneous Melanoma / Cutaneous Melanoma; COAD: Colorectal Cancer/Colon Adenocarcinoma; CRC: Colorectal Cancer/Colorectal Cancer; DLBCL: Lymphoma/Diffuse Large B‐cell Lymphoma; GBM: Brain Tumour / Glioblastoma; Glioma: Brain Tumour/Glioma; GEC: Gastroesophageal Cancer/Gastroesophageal Cancer; HER2‐BRCA: Breast Cancer/HER2 Breast Cancer; HGSOC: Ovarian Cancer/High‐Grade Serous Ovarian Cancer; LCA: Lung Cancer/Lung Cancer; National Lung Screening Trial (NLST); LSCC: Lung Cancer/Squamous Cell Carcinoma; LUAD: Lung Cancer/Adenocarcinoma; PCA: Prostate Cancer/Prostate Cancer; PDA: Pancreatic Cancer/Pancreatic Ductal Adenocarcinoma; SAR: Sarcoma/Sarcoma; SLN‐BRCA: Breast Cancer Axillary Lymph Node Metastases/Axillary Lymph Node Metastases; UCEC: Uterine Corpus Endometrial Carcinoma/Uterine Corpus Endometrial Carcinoma. Details are presented in Table S1.

To investigate cellular architecture across solid tumours, we assembled a large cohort of H&E‐stained WSIs from the TCIA database.^9^ The dataset included over 10 000 WSIs spanning multiple cancer types, such as breast cancer (BRCA,^10^, ^11^ HER2‐BRCA,^12^ SLN‐BRCA^13^), kidney cancer (CCRCC,^14^ Non‐CCRCC^14^), melanoma (CM^15^), colorectal cancer (COAD,^16^ CRC^17^), lymphoma (DLBC^18^), brain tumours (GBM,^19^, ^20^ Glioma^21^), gastric cancer (GEC^22^), ovarian cancer (HGSOC^23^), lung cancer (LCA,^24^ NLST,^25^ LSCC,^26^ LUAD^27^), prostate cancer (PCA^28^), pancreatic cancer (PDA^29^), sarcoma (SAR^30^) and uterine cancer (UCEC^31^). Full abbreviations are provided in the Figure 1A (footnote). These tumours were grouped by both histology and data source. For instance, the NLST dataset^25^ is classified as lung cancer by histology, but its distinct origin offers an additional layer for comparison.

To obtain these measurements, we leveraged a deep learning‐based segmentation workflow^32^, ^33^ to identify cell nuclei from H&E‐stained WSIs (Figure 1B). We segmented nuclei without artificial cytoplasm expansion, with original boundaries shown in red for representative samples from each cancer type (Figure 1B). After quality control, 7910 WSIs were retained for further analysis (Figure 1C). In total, this yielded a dataset of over 4.7 billion cells (4 704 990 971) with precise spatial coordinates, derived from a tissue area of more than 730 billion square micrometres (730 682 190 578 µm^2^) (Figure 1C).

In summary, we implemented an integrated and scalable analytical pipeline to explore cellular architecture in solid tumours. By providing single‐cell resolution coordinates, it enables downstream analyses aimed at understanding structural differences across diverse cancer types.

### 2.2 Establishing nearest neighbour distance as a metric for cellular architecture

**FIGURE 2:**
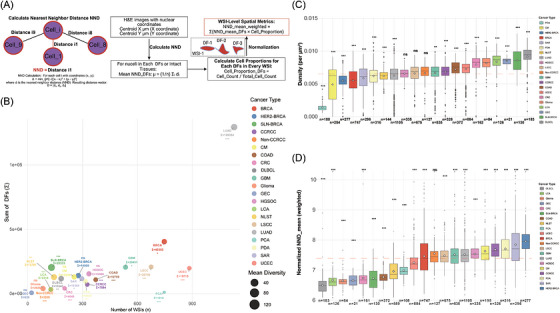
NND variation arises from solid tumour heterogeneity. (A) The diagram illustrated the spatial calculation of the NND. An NND value was assigned to each cell within the tissue. For each DF, a representative NND value was calculated as the mean of all cellular NND values within that region. Finally, the WSI‐level NND value was derived by computing the weighted mean of all DF‐level values, with weights corresponding to the cellular proportion of each DF. (B) The bubble plot visualized the aggregate number of DFs per cancer type. (C) The box plot illustrated the distribution of cell density across different cancer types. (D) The box plot showed the variation in WSI‐level NND values (normalized) among the cancer types analysed. Statistical significance was assessed for each solid tumour type using a per‐sample *t*‐test against the overall mean, with FDR correction applied. Significance levels were set at *p < .05, **p < .01, and ***p < .001 (FDR‐adjusted). Non‐significant results (*p* > .05) are not denoted. Details are indicated in Table S2.  NND, Nearest Neighbour Distance; DF, disconnected tissue fragment; WSI, whole‐slide image.

To systematically compare spatial cellular differences across solid tumours, we introduced NND as a key metric for every cell in our cohort (Figure 2A). For a given cell, NND is defined as the distance to its closest neighbour. To generate a WSI‐level summary, we computed a normalized NND value by taking a weighted mean of these single‐cell distances, with weights proportional to the number of cells within each discontinuous tissue fragment (DF) (Figure 2A). This approach addresses the challenge of tissue fragmentation commonly seen in clinical slides.

Across the tumour types analysed, LUAD showed the highest degree of fragmentation with 129 354 DFs, followed by BRCA with 40 353 DFs, highlighting substantial variation in tissue architecture (Figure 2B). We then compared overall cell density and normalized NND across tumour types (Figure 2C,D). These results revealed both similarities and distinct differences for cellular architecture. In terms of cell density, DLBCL, SLN‐BRCA, GEC and LCA ranked highest (Figure 2C). For normalized NND, DLBCL, LCA, Glioma and GEC exhibited the lowest values (Figure 2D). While high density generally corresponded with low NND in most cases, as expected, notable exceptions emerged. For instance, SLN‐BRCA exhibited high cell density yet did not show proportionally low NND values in the ranking (Figure 2C,D), suggesting a distinct spatial configuration where cells are numerous but not as tightly packed (Figure 2D). Conversely, Glioma displayed low NND despite not ranking among the highest‐density tumours, pointing to localized clustering rather than uniformly dense packing (Figure 2D). These patterns reveal that tissue organization in specific tumours follows non‐random spatial logics that go beyond simple density‐NND relationships.

We also examined the relationship between NND and cell density across tumours (Figure S1A).

Strong negative correlations (*ρ* < ‐.9, FDR *p* < .001) were observed in SLN‐BRCA and PDA. This tight coupling between density and packing indicates a highly homogeneous cellular distribution within these tumour microenvironments, where densely packed regions consistently show proportionally smaller intercellular distances. In contrast, most other tumours exhibited only moderate correlations (*ρ* = .7‐.8, FDR *p* < .001). This weaker association points to a more regionally aggregated or heterogeneous spatial architecture, where local variations in tissue organization decouple density from NNDs.

In summary, by introducing and applying the normalized NND metric alongside conventional cell density analysis, we quantified distinct spatial architectures across 21 solid tumour types. While cell density reflects the overall cellularity within the tissue, NND captures local spatial organization, revealing patterns of cellular clustering and packing that density alone cannot resolve. For example, in cancer types with weak density‐NND correlations (GBM, PCA, HGSOC, LUAD), NND revealed local crowding features that were not apparent from density measurements alone (Figure S1A,B). This work establishes NND as a quantitative metric of regional cellular aggregation directly extracted from routine H&E‐stained WSIs. These observations suggest that NND may serve as a complementary spatial metric worthy of further investigation as a potential image‐based biomarker.

### 2.3 Low NND associates with higher mechanical Hippo/YAP/TAZ pathway activity and CD4^+^T_Mem_ cell fraction

**FIGURE 3:**
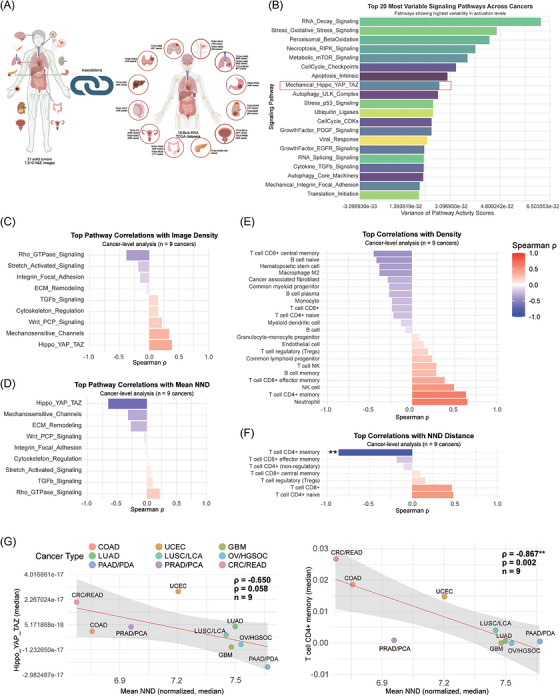
Low NND associates with higher Mechanical Hippo/YAP/TAZ pathway activity and CD4^+^T_Mem_ cell fraction. (A) Study cohort composition. Schematic overview of the pan‐cancer cohorts used for integrative analysis. The study included bulk RNA‐seq data from 19 TCGA solid tumour types and H&E‐stained histopathological slides from 21 solid tumour types. Matched cancers between the two cohorts were utilized to investigate the association between image‐derived morphological phenotypes and transcriptomic molecular features across cancer types. (B) Bar plot displaying the scores of the top 20 most variable signalling pathways across the analysed cancers. (C and D): Heatmaps of signalling pathways associated with tumour microenvironment features. Selected top 15 signalling pathways correlated with (C) overall cell density and (D) normalized NND across 9 cancer types. Pathways were ranked by the absolute Spearman's *ρ* coefficient. *p*‐values approaching .05 were labeled with their exact values. E‐F. Heatmaps of correlations between spatial metrics and immune cell composition. (E) Top correlations of overall cell density with deconvoluted cell type fractions. (F) Top correlations of NND with deconvoluted cell type fractions predicted from bulk RNA expression. The top correlations were jointly selected based on their association strength with both overall cell density and NND metrics. (G) Scatter plots show the association of normalized NND with Hippo/YAP/TAZ pathway activity (left) and CD4^+^ memory T cell abundance (right). Each data point represents one of the nine histologically matched cancer types: COAD, CRC/READ, GBM, OV/HGSOC, LSCC/LCA, LUAD, PRAD/PCA, PAAD/PDA and UCEC. Correlation analysis was performed using Spearman's method (*n* = 9). For NND vs. Hippo/YAP/TAZ: Spearman's *ρ* = ‐.65, *p* = .058; for NND correlated with CD4^+^ memory T cell: Spearman's *ρ* = ‐.86, *p* < 0.01. Ovarian serous cystadenocarcinoma (TCGA‐OV); Rectum adenocarcinoma (TCGA‐READ); Esophageal carcinoma (TCGA‐ESCA); Head and Neck squamous cell carcinoma (TCGA‐HNSC); Lung squamous cell carcinoma (TCGA‐LUSC); Testicular germ cell tumours (TCGA‐TGCT); Pancreatic adenocarcinoma (TCGA‐PAAD); Stomach adenocarcinoma (TCGA‐STAD); Kidney renal papillary cell carcinoma (TCGA‐KIRP); Thyroid carcinoma (TCGA‐THCA); Colon adenocarcinoma (TCGA‐COAD); Uterine corpus endometrial carcinoma (TCGA‐UCEC); Prostate adenocarcinoma (TCGA‐PRAD); Bladder urothelial carcinoma (TCGA‐BLCA); Lung adenocarcinoma (TCGA‐LUAD); Mesothelioma (TCGA‐MESO); Glioblastoma multiforme (TCGA‐GBM); Liver hepatocellular carcinoma (TCGA‐LIHC); Pheochromocytoma and Paraganglioma (TCGA‐PCPG); The Cancer Genome Atlas (TCGA), Nearest Neighbour Distance (NND); False Discovery Rate (FDR). Details are shown in Table S3.

Motivated by the pronounced heterogeneity of NND observed across solid tumours, we hypothesized that high cellular density in tissue regions may elevate mechanical stress within TME. To investigate the associations of overall cell density and NND with immune cell composition and signalling activities, we leveraged bulk RNA‑seq data from The Cancer Genome Atlas (TCGA). Following quality control, 19 solid tumour types were retained for deconvolution analysis, enabling simultaneous estimation of signalling pathway activity and TME cell‑type abundances (Figure 3A).

Across these tumours, we identified the most prominently activated signalling pathways including RNA decay and oxidative stress, with an aggregated analysis of activation scores further highlighting the Mechanical/Hippo/YAP/TAZ pathway as a top‐ranking feature (Figure 3B).

We then constructed an integrative model to correlate overall cell density and NND with pathway activities, stratifying the analysis by the median value of each cancer type. Nine cancer types were retained for evaluation following histology‐based matching between the H&E whole‐slide cohort and the TCGA bulk RNA‑seq solid tumour dataset. Among the top rank pathways ranked by correlation strength, overall cell density showed no statistically significant associations (Figure 3C). A trend‐level association was observed between lower NND and higher Hippo/YAP/TAZ pathway activity (Spearman's *ρ* = ‐.65, *p* = .058, *n* = 9; raw data provided in Supporting Information [Tables]). This correlation did not reach conventional statistical significance, in part due to the limited sample size (*n* = 9) inherent to cross‐cohort aggregation. In contrast, the association between lower NND and higher CD4^+^T_Mem_ cell abundance reached statistical significance (Spearman's *ρ* = ‐.86, *p* < .01) (Figure 3D,G). These results suggest that mechanical stress in these solid tumours is reflected by NND values, further supporting the use of NND as a spatial biomarker for evaluating tumour mechanical stress.

To infer the cellular composition of the TME, we performed deconvolution analysis^32^, ^33^, ^34^, ^35^ on bulk RNA‑seq data using cell type‑specific gene signatures, obtaining relative abundances of TME cell populations across solid tumours (Figure S2A). Based on these algorithms, deconvolution analysis resolved the cellular composition of the TME, identifying CD4^+^ Th2 T‐cell (Figures S2A; Figure S3A), activated myeloid dendritic cells (Figure S2A; Figure S3B), hematopoietic stem cells (Figure S2A; Figure S3C), memory B cells, cancer‑associated fibroblasts, endothelial cells, macrophages, Nature killer (NK)‐T‐cell and CD4^+^ Th1 T‐cell among the most abundant cell types (Figure S2A).

We then evaluated the correlations of overall cell density and NND with each TME cell type fraction. To prioritize the most robust associations, we pooled the correlation strengths from both spatial metrics and selected the top‑ranked relationships for downstream analysis (Figure 3E,F). Overall cell density showed no significant correlations with most TME cell fractions. CD4^+^T_Mem_ cells and neutrophils were the only exceptions, each displaying moderate positive associations (absolute Spearman's *ρ* > .5, *p* > .05) (Figure 3E). In contrast, spatial analysis revealed that the only significant correlation observed was a negative one between CD4^+^T_Mem_ cells and NND (absolute Spearman's *ρ* > .8, *p* < .01) (Figure 3F,G). More specifically, COAD and CRC exhibited lower NND values, whereas LUAD, LUSC, GBM, OV and PAAD showed higher NND accompanied by lower Hippo/YAP/TAZ pathway activity and reduced CD4^+^T_Mem_ cell fractions (Figure 3G; Figure S3D). These results indicate that CD4^+^ TMem cells show enrichment in regions associated with high mechanical stress, as exemplified by the low‐NND, high‐stress microenvironment of CRC or COAD.

To provide a holistic view of the TME, we calculated a composite score based on immune and stroma signatures across the TCGA solid tumours (Figure S2B). Immune and stromal scores were inversely correlated. Most strikingly, TCGA‐LIHC and TCGA‐PCPG showed the highest stromal scores, pointing to a distinct stroma‐immune turnover phenotype that deserves deeper exploration (Figure S2B). We then integrated overall cell density and NND to assess their associations with the composite immune and stroma score (xCell)^33^ across the nine matched solid tumours (Figure S2C). Overall cell density showed no significant correlations (absolute Spearman's *ρ* < .5) (Figure S2C). In contrast, NND was positively and significantly correlated with both the immune infiltration score(xCell)^33^ (Spearman's *ρ* > .5, *p* < .05), though not with the stromal score (Figure S2C). These results may indicate that immune cell infiltration may require a certain degree of spatial freedom (reflected by NND) to facilitate intercellular interactions, a mechanistic insight that warrants further experimental investigation.

Given the above results, we propose that NND serves as a potentially informative spatial parameter for evaluating mechanical stress, based on its trend‐level associations with Hippo/YAP/TAZ signalling and significant associations with CD4^+^ TMem cell abundance.

### 2.4 Single‐Cell landscape of T cell microenvironmental niches and mechanical signalling

validate the link between low NND, high mechanical stress, and CD4^+^T_Mem_ cells, we screened more than five GEO cancer single‐cell datasets. These comprised over 300 000 cells in total. After stringent T cell‐focused quality control, five datasets were retained for downstream analysis. These included melanoma (GSE123813; 53 780 cells),^36^ colorectal cancer (GSE178341; 9295 cells),^37^ hepatocellular carcinoma (GSE149614; 2534 cells),^38^, ^39^ intrahepatic cholangiocarcinoma (GSE138709; 299 cells),^40^ and intestinal‐type gastric cancer (GSE134520; 255 cells)^41^ (Figure S4A).

**FIGURE 4:**
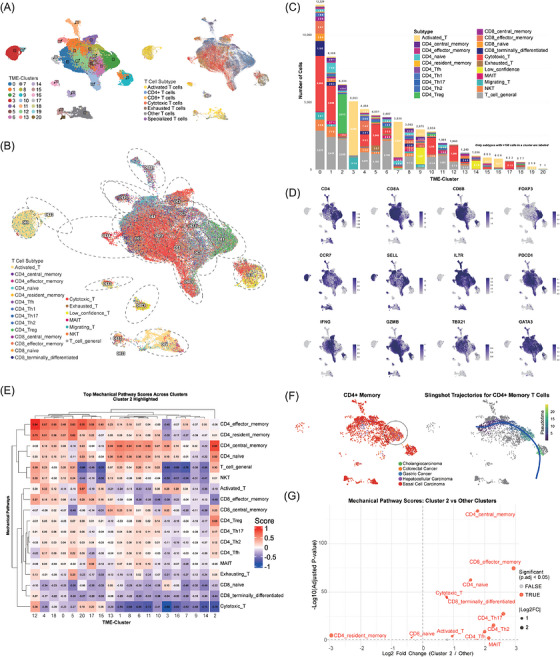
Single‐cell landscape of T Cell microenvironmental niches and mechanical signalling. (A) UMAP visualization of 21 microenvironmental clusters (TME‐clusters) identified through unsupervised clustering of integrated scRNA‐seq data from five public datasets (GSE123813, GSE134520, GSE138709, GSE149614, GSE178341). TME‐clusters were defined based on shared microenvironmental signatures and ecological niche similarity rather than lineage alone. Each dot represents a single cell, coloured by cluster identity. The UMAP projection also highlights major lineage categories, including CD4^+^ T‐cells, CD8^+^ T‐cells, regulatory T‐cells, helper T‐cells, specialized T‐cells, memory T‐cells, exhausted T‐cells, activated T‐cells, cytotoxic T‐cells and other T‐cells. (B) UMAP visualization of T‐cells coloured by detailed subtype annotation, providing high‐resolution mapping of populations including Activated_T, CD4_naive, CD4_central_memory, CD4_effector_memory, CD4_resident_memory, CD4_Tfh, CD4_Th1, CD4_Th2, CD4_Th17, CD4_Treg, CD8_naive, CD8_central_memory, CD8_effector_memory, CD8_terminally_differentiated, Cytotoxic_T, Exhausted_T, MAIT, Migrating_T, T_cell_general, and Low_confidence_T‐cells. (C) Stacked bar plot showing the percentage composition of T cell subtypes within each of the 21 microenvironmental clusters. This distribution reflects how different TME niches recruit or induces specific T cell populations. Numbers above bars indicate total cell count per cluster; only subtypes comprising > 5% of a cluster are labelled. (D) UMAP visualization of key lineage and functional markers for T‐cell identification, with emphasis on CD4^+^ memory T cell‐defining markers. Expression levels are shown for Pan‐T (CD4, CD8A, CD8B); Regulatory (FOXP3); Naïve/Memory (CCR7, SELL, IL7R); Exhaustion (PDCD1); Effector (IFNG, GZMB); and Master Regulators (TBX21, GATA3). (E) Heatmap of mechanical pathway activity across T‐cell subtypes and microenvironmental clusters. Elevated mechanical signalling activity is particularly prominent in Cluster 2, indicating a high‐pressure niche. F: UMAP projection illustrating the tissue origin and developmental trajectory of CD4^+^T_Mem_ cells. Cells are coloured by dataset/sample type, showing a primary derivation from melanoma and colorectal cancer. Notably, the Cluster 2 niche exhibits a higher relative abundance of CD4^+^T_Mem_ cells from colorectal cancer compared to other clusters. Overlaid lines represent Slingshot trajectory analysis, revealing a progressive differentiation path of CD4^+^ T cells from Cluster 0 (naïve, root) toward Clusters 2 and 9 (memory states). Cluster 19 was omitted owing to not enough subsets of T‐cells. (G) Volcano plots comparing mechanical pathway scores in Cluster 2 versus all other clusters. Each point represents a T‐cell subtype, highlighting those with the most significantly elevated scores within this specific niche. Horizontal dashed lines indicate the significance threshold (adjusted *p*‐value < .05), and vertical dashed lines indicate the absolute log^2^ fold‐change threshold. Gene signatures for the mechanical stress are available in Table S4.  CD4^+^T_Mem_ cell, CD4^+^ memory T‐cells.

Instead of clustering cells solely by their lineage‐specific lineage markers, we performed unsupervised clustering to group T‐cells based on their microenvironmental similarity and shared ecological niches. This approach revealed 21 distinct clusters reflecting diverse functional states shaped by the TME (Figure 4A,B). We then performed phenotypic profiling to define subtype identities within these environmentally‐defined clusters. Cytotoxic/effector T cells were defined by expression of CD8A and GZMB (this population predominantly comprises activated CD8^+^ T cells with cytotoxic potential). Tregs were identified by CD4 and FOXP3 expression. CD4^+^ T cells were divided into multiple distinct subsets: CD4^+^ naive T cells (expressing CCR7, SELL, TCF7), these are NOT memory T cells; CD4^+^ central memory T cells (expressing CCR7, SELL, IL7R); CD4^+^ effector memory T cells (expressing GZMB and IL7R, lacking CCR7/SELL); and CD4^+^ resident memory T cells (expressing ITGAE and CXCR6). The term ‘CD4^+^_TMem_ cells’ in this study refers exclusively to central memory, effector memory and resident memory CD4^+^ T cells, excluding naive CD4^+^ T cells (Figure S4B,C).

The distribution of these subtypes across clusters highlighted the intense heterogeneity of the TME, where each cluster represents a unique mechanical and biochemical “neighbourhood”. Cluster 0 contained the highest number of T‐cells, with 12 239 cells (Figure 4C), among which cytotoxic T‐cells accounted for 4056 cells (33.2%) (Figure 4C; Figure S4D). In contrast, Cluster 2 represented a niche with the highest number of CD4^+^ Tregs, with 2913 cells (46.0%) (Figure 4C; Figure S4D). Cluster 3 showed the highest number of activated T‐cells, with 4029 cells (79.6%). This analysis showed that each cluster contained different proportions of T‐cell subtypes (Figure S4D), reflecting how varying microenvironmental pressures recruit or induce distinct T‐cell populations within the TME.

To further characterize these niches, we analysed key lineage and functional markers across clusters, including pan‐T‐cell markers (CD4, CD8A, CD8B), regulatory markers (FOXP3), naïve and memory markers (CCR7, SELL, IL7R), exhaustion markers (PDCD1), effector markers (IFNG, GZMB), and master regulators (TBX21 for Th1, GATA3 for Th2) (Figure 4D).

We then highlighted Cluster 2 for its mechanical pathway scores to show the enrichment of mechanical signalling pathways across these microenvironmental clusters and T‐cell subtypes (Figure 4E). When we investigated mechanical signalling pathways, we found that mechanical pathway‐related genes were specifically enriched in niches dominated by CD4^+^ T‐cell populations, particularly CD4^+^T_Mem_ cells (Figure 4E).

The enrichment patterns differed across clusters, indicating diverse mechanical adaptation strategies. In Clusters 2, 13, 1, 8, 6, and 11, CD4^+^ central memory T‐cells and CD4^+^ naïve T‐cells showed the highest enrichment of mechanical signalling pathways (Figure 4E). In other clusters, the enrichment was most pronounced in different CD4^+^ T_Mem_ subsets: CD4^+^ effector memory T‐cells showed strong enrichment in Clusters 4, 5, 12, 15, 17, 18 and 20, while CD4^+^ resident memory T‐cells exhibited the highest enrichment in Clusters 0, 4, 5, 12 and 18 (Figure 4E). These results indicate that CD4^+^ T_Mem_ cells are the primary populations associated with mechanical signalling niches, highlighting their association with mechanical stress and adaptation to the physical TME.

We next investigated the tumour‐type origins of CD4^+^ T_Mem_ cells, which were mostly derived from melanoma (Figure 4F). Pseudo‐temporal trajectory analysis on CD4^+^ T_Mem_ cells across all clusters revealed a transition trajectory toward Cluster 2 and Cluster 9 (Figure 4F). These results indicate a developmental relationship among these CD4^+^ T_Mem_ cells, with Cluster 9 representing a later state along the trajectory.

By combining these trajectory findings with the mechanical signalling analysis, we observed distinct patterns of environmental adaptation. CD4^+^ T_Mem_ cells in earlier states (e.g., Cluster 12) expressed higher levels of mechanical signalling pathways than those in later states, such as Cluster 9 (Figure 4E,F). This was particularly evident for CD4^+^ effector memory T‐cells and CD4^+^ resident memory T‐cells (Figure 4E,F). By comparison, CD4^+^ central memory T‐cells and CD4^+^ naïve T‐cells showed higher mechanical signalling pathway enrichment as they transitioned into later‐state niches, such as Cluster 2 (Figure 4E,F). Within Cluster 2 specifically, mechanical signalling pathways were most significantly enriched in central memory CD4^+^ T‐cells compared to other T cell subtypes (Figure 4G).

Together, these results validated the enrichment of mechanical signalling pathways in CD4^+^ T_Mem_ cells based on their microenvironmental context. This confirmed a robust link between NND, CD4^+^ T_Mem_ cells, and the mechanical signalling axis.

### 2.5 Spatial transcriptomic validation of mechanical pathway activity in a low‐nnd cancer type (Colon Cancer)

**FIGURE 5:**
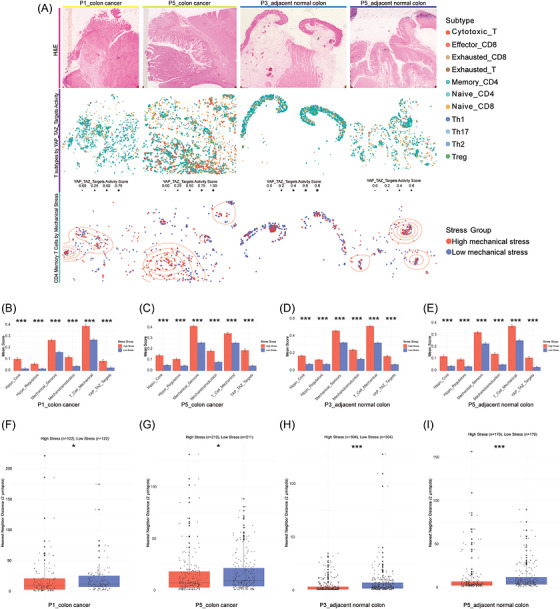
Spatial transcriptomic analysis of CD4^+^ T_Mem_ cells in colon cancer. (A) High‐resolution spatial mapping of mechanical signalling in T‐cell subtypes and CD4+ memory T‐cells. Tissue sections from colon cancer (P1, P5) and normal colon (P3, P5) samples were analysed using Visium‐HD spatial transcriptomics. Pathway activity scores are projected onto H&E‐stained images to visualize the distribution of (i) YAP/TAZ target activity in T‐cell subsets and (ii) differential mechanical stress responses (high/low) in CD4+ memory T‐cells within the tissue microenvironment. High and low mechanical stress groups were defined using the median of the same mechanical gene signature. Comparison between groups therefore reflects a coherent expression gradient. Circled regions denote areas of T‐cell crowding and low NND values. (B–E): Comparison of mechanical pathway scores in CD4^+^ T_Mem_ cells. Box plots showing Hippo/YAP/TAZ pathway score and mechanical signalling pathway score in CD4^+^ T_Mem_ cells from cancer (P1, P5) and normal (P3, P5) tissue sections. Spots were stratified into high and low mechanical stress groups based on median pathway activity. Colour schemes are consistent with those used for each subtype throughout the figure. (F–I) NND comparison CD4^+^ T_Mem_ cells under differential mechanical stress. Box plots comparing nearest neighbour distances (NND) between high and low mechanical stress CD4^+^ memory T cells from colon cancer (P1, P5) and adjacent normal (P3, P5) tissue sections. Spots were stratified based on median composite mechanical pathway activity. NND was calculated as the minimum distance to the nearest neighbouring CD4^+^ memory T cell (2 µm/spots). Statistical significance with *p*‐values shown. High stress spots (red) showed decreased NND compared to low stress spots (blue) in of samples, indicating clustered spatial organization under high mechanical stress conditions. See Table S5 for details.  CD4^+^ T_Mem_ cell, CD4^+^ memory T‐cells; nearest neighbour distances (NND).

To further investigate whether mechanical stress is enriched in CD4^+^ T_Mem_ cells within low NND regions, we performed high‐resolution spatial transcriptomics (Visium HD, 2 µm spot resolution) on matched cancer and normal tissue sections (Figure  5A).^42^

We first resolved T‐cell subtypes in these tissues. CD4^+^ T_Mem_ cells showed higher YAP/TAZ signalling scores, particularly in T‐cell aggregation areas where multiple T‐cell subtypes were enriched (Figure  5A). Using the mechanical gene signature (see Methods), CD4^+^ T_Mem_ cells were stratified into high and low mechanical stress groups based on the median pathway score within each tissue section. The same signature was then used to compare expression levels between the two groups. The high mechanical stress group showed higher expression of mechanical signalling pathways in T‐cell aggregation areas (Figure  5A). This analysis demonstrates a coherent expression gradient along the defined mechanical stress axis rather than providing independent validation.

This pattern was observed in both cancer and normal tissues. Multiple mechanical signalling pathways were significantly upregulated in the high stress group compared to the low stress group (Figure 5B–E). These included Hippo_Core, Hippo_Regulators, Mechanical_Sensors, Mechanotransduction, T_Cell_Mechanical and YAP_TAZ_Targets.

Together, these spatial transcriptomics results confirm that CD4^+^ T_Mem_ cells in T‐cell aggregation regions exhibit elevated mechanical signalling pathway activity. These aggregation regions are consistent with low NND areas, further supporting the link between NND‐defined mechanical stress and the CD4^+^ T_Mem_ cell phenotype. NND analyses revealed that CD4^+^ T_Mem_ cells under high mechanical stress exhibited significantly decreased NND compared to their low‐stress counterparts (Figure 5F–I), indicating a more clustered spatial organization under conditions of elevated mechanical signalling. This pattern was consistently observed across both cancer and adjacent samples (*p* < .05), suggesting that mechanical stress associated with cell–cell proximity or regional aggregation of CD4^+^ T_Mem_ cells within the tissue microenvironment.

### 2.6 Prognostic impact of mechanical pathway activity and CD4^+^T_Mem_ cell infiltration in solid tumours

**FIGURE 6:**
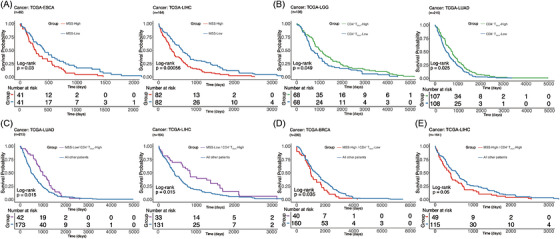
Prognostic impact of mechanical pathway activity and CD4^+^ T_Mem_ cell infiltration in solid tumours. (A) KM survival analysis for tumours stratified by MSS alone (median cutoff: high vs. low) in TCGA‐ESCA and TCGA‐LIHC. (B) KM survival analysis for tumours stratified by CD4^+^ T_Mem_ infiltration level alone (median cutoff: high vs. low) in TCGA‐LGG and TCGA‐LUAD. (C) KM survival analysis of TCGA‐LUAD and TCGA‐LIHC patients jointly stratified by both features. Group 1: MSS low / CD4^+^ T_Mem_ high; Group 2: all other patients. (D) KM survival analysis of TCGA‐BRCA patients jointly stratified by both features. Group 1: MSS high / CD4^+^ T_Mem_ low; Group 2: all other patients. (E) KM survival analysis of TCGA‐LIHC patients jointly stratified by both features. Group 1: Double‐High (MSS high / CD4^+^ T_Mem_ high); Group 2: all other patients. For dual‐factor survival analyses (C–E), patients within each cancer type were first stratified into four subgroups based on median cutoffs of both MSS and CD4^+^T_Mem_ score: Double‐High (MSS high / CD4^+^T_Mem_ high), Double‐Low (MSS low / CD4^+^T_Mem_ low), MSS high / CD4^+^T_Mem_ low, and MSS low / CD4^+^T_Mem_ high. For each KM comparison, the indicated subgroup (Group 1) was compared against all other patients combined as Group 2 (the remaining three subgroups). This approach prioritizes statistical power, with the limitation that the reference group is heterogeneous in biological composition. Detailed information are available in Table S6. Mechanical Signalling Score (MSS); CD4^+^ memory T‐cells, CD4^+^ T_Mem_ cell;The Cancer Genome Atlas (TCGA); Esophageal carcinoma (ESCA); Lung adenocarcinoma (LUAD); Liver hepatocellular carcinoma (LIHC); Brain Lower Grade Glioma (LGG); Breast Invasive Carcinoma (BRCA); Kaplan–Meier (KM).

Building upon the identified NND‐CD4^+^ T_Mem_ cells ‐mechanical signalling axis, we evaluated its prognostic impact across 21 TCGA bulk‑RNA sequencing datasets^43^ After prioritizing datasets with robust clinical follow‐up and sufficient sample sizes, we focussed on cohorts showing significant survival associations (Figure 6A–E).

Kaplan–Meier analysis first established the independent prognostic value of these signatures. High mechanical signalling scores were significantly correlated with poorer overall survival (OS) in TCGA‐ESCA and TCGA‐LIHC (*p* < .05; Figure 6A). In contrast, an elevated CD4^+^T_mem_ T‐cell signature generally predicted more favourable outcomes (*p* < .05; Figure 6B).

Integrating these two metrics further revealed that their interaction dictates distinct survival patterns across cancer types. In TCGA‐LUAD and TCGA‐LIHC, patients with a ‘Low Mechanical/High CD4^+^T_Mem_ cell’ profile experienced the most favourable survival (Figure 6C). Conversely, the ‘High Mechanical/Low CD4^+^ T_Mem_ cell’ phenotype was associated with the worst prognosis in TCGA‐BRCA (Figure 6D). Notably, in TCGA‐LIHC, even the ‘Double‐High’ subgroup (high mechanical/high CD4^+^ T_Mem_ cell) showed a survival benefit, suggesting a complex interplay in certain microenvironments (Figure 6E).

Together, these findings demonstrate that mechanical signalling is a potent prognostic determinant, and its integration with CD4^+^T_Mem_ cell infiltration provides a more nuanced framework for predicting patient outcomes across multiple solid tumours.

## 3 DISCUSSION

Our findings suggest that NND captured aspects of tumour architecture distinct from conventional cell density measurements. Density reflected overall cellularity. In contrast, NND emphasized local cell spatial organization, revealing patterns of cell aggregation that density alone could not resolve.

The dissociation between these metrics in specific cancer types demonstrated that tumours followed non‐random spatial logics. For example, SLN‐BRCA exhibited high density without proportionally low NND. These patterns may reflect underlying biological processes. And we further uncovered a conserved axis associating high cellular aggregation (low NND) to activation of mechanical pathways and enrichment of CD4^+^ T_Mem_ cells.

Across nine histology‐matched cancer types, we linked the H&E cohort with TCGA bulk RNA‐seq data with validation across bulk transcriptomic deconvolution. A trend‐level association was observed between lower NND and higher Hippo/YAP/TAZ pathway activity (*ρ* = ‐.65, *p* = .058, *n* = 9), a canonical mechano‐transduction cascade. This correlation did not reach conventional statistical significance, in part due to the limited sample size (*n* = 9) inherent to cross‐cohort aggregation. This association positioned NND as a spatially derived proxy for mechanical stress that could be extracted directly from routine pathology slides. Notably, overall cell density failed to show comparable correlations, underscoring the importance of local spatial organization as a determinant of tissue biomechanics. Further analysis revealed that low NND also significantly correlated with CD4^+^ T_Mem_ cell enrichment.

Several limitations regarding the interpretation of NND as a mechanical stress proxy should be noted. NND is a geometric descriptor, not a direct measurement of mechanical force. While low NND is broadly associated with higher mechanical crowding as a well‐established principle in tissue biomechanics, identical NND values in different tissues or cancer types may correspond to different absolute mechanical stresses due to variations in extracellular matrix density, fibre alignment, nuclear‐to‐cytoplasmic ratio, and cell‐matrix adhesion properties. Therefore, NND is best interpreted as a relative spatial metric within histologically comparable groups rather than a calibrated, cross‐tissue absolute measure of mechanical force. No direct biomechanical measurements, such as tissue stiffness or ECM properties, were performed in this study. Furthermore, NND does not distinguish neighbour cell identity, and the observed associations may be influenced by tumour purity, immune infiltration, and other compositional factors.

Furthermore, by integrating single‐cell RNA‐seq data from multiple solid tumours, we uncovered that CD4^+^ T_Mem_ cells preferentially expressed mechanical signalling pathways. This enrichment was specific to CD4^+^ T_Mem_ cell subsets, particularly central memory, effector memory, and resident memory populations. These results suggested that such CD4^+^ T_Mem_ cells may be uniquely associated with sense and respond to physical cues within a crowded TME. Pseudo‐temporal trajectory analysis further indicated that mechanical pathway activity varied with CD4^+^ T_Mem_ cell differentiation state, with earlier memory states such as central memory and effector memory showing higher mechano‐signalling enrichment. This raised the possibility that mechanical stress within the TME actively correlates with the phenotype and positioning of CD4^+^ T_Mem_ cell, with potential implications for the durability of anti‐tumour immune responses. A limitation of our single‐cell analyses is the absence of a negative control dataset, such as T cells from in vitro culture or PBMCs, that would allow baseline mechanical signalling levels to be assessed in the absence of tissue crowding.

The spatial transcriptomics data provided direct visualization of this phenomenon, demonstrating that CD4^+^ T_Mem_ cells within T cell aggregation areas, corresponding to low‐NND regions, exhibited elevated expression of mechanotransduction genes. That this pattern was observed in both cancer and normal colon tissues suggested that the coupling between tissue crowding and CD4^+^ T_Mem_ cell mechanosensing may reflect a fundamental aspect of tissue immune physiology, potentially co‐opted or amplified during tumorigenesis. However, the spatial transcriptomics validation was restricted to colon cancer samples only (two cancer and two adjacent normal tissues from the Visium‐HD dataset). Validation in additional tumour types (liver, lung, breast) is needed to assess the pan‐cancer generalizability of the observed spatial‐mechanical‐immune axis. Furthermore, current spot‐based spatial transcriptomics, such as Visium HD at 2 µm resolution, does not permit direct single‐cell NND calculation or comprehensive cell‐cell communication analysis. Future studies using higher‐resolution platforms, such as Xenium, MERFISH, or multiplexed imaging, with true single‐cell resolution will be essential to directly measure intercellular distances and dissect the molecular crosstalk within mechanically constrained niches. Clinically, this spatial‐mechanical‐immune axis carried prognostic significance. High mechanical signalling scores were associated with poorer survival in oesophageal and liver cancers, while CD4^+^ T_Mem_ cell signatures correlated with favourable outcomes. The combinatorial analysis revealed that patients with low mechanical signalling accompanied by high CD4^+^ T_Mem_ cell infiltration experienced the most favourable survival in lung adenocarcinoma and liver cancer. Conversely, the high mechanical/low CD4^+^ T_Mem_ cell profile identified a poor‐prognosis subgroup in breast cancer. Interestingly, a ‘double‐high’ profile was also associated with beneficial in liver cancer, indicating that the functional state of infiltrating CD4^+^ T_Mem_ cells, possibly activated and supported by mechanical signalling, may be crucial. The divergent survival patterns between LIHC and BRCA suggest context‐dependent effects of mechanical signalling on CD4^+^ T_Mem_ cells. In LIHC, the native mechano‐adaptive environment of the liver might be associated with mechanical crowding enhancing T cell function rather than suppressing it. In contrast, the dense desmoplastic stroma of breast cancer may render mechanical stress predominantly detrimental to T cell infiltration or activity. Alternatively, the double‐high profile in LIHC may simply reflect a general state of immune activation. These hypotheses require functional validation but underscore the importance of tissue context in interpreting the mechanical‐immune axis.

In the dual‐factor survival analyses (Figure 6C–E), each specific subgroup (such as Mechanical Low / CD4 High) was compared against ‘all other patients combined’ (as the remaining three subgroups). This approach prioritizes statistical power by maximizing sample size in the reference group, but it comes with the limitation that the reference group is heterogeneous in biological composition. Additional limitations should be noted as no patient‐level pairing exists between our WSI cohort (TCIA) and the TCGA bulk RNA‐seq cohort. The correlation analysis was performed at the cancer type level (*n* = 9) using median values per cancer type, not at the individual patient level. This aggregation was necessary because the two cohorts are independent and non‐overlapping. The limited sample size (*n* = 9) provides low statistical power; therefore, the cancer‐type level correlations are hypothesis‐generating only and should be interpreted with caution.

Despite these advances, several limitations of this study should be noted. First, NND captured nuclear positions but did not directly measure mechanical forces. Future studies incorporating direct biophysical measurements will be necessary to calibrate NND against absolute stress values. Second, integrating spatial metrics with bulk transcriptomics required histological matching between cohorts, which may obscure intratumoral heterogeneity. Third, while our analyses supported the association between mechanical signalling and CD4^+^ T_Mem_ cells, functional studies using 3D culture systems or murine models are needed to establish causality. Fourth, the prognostic models require validation in independent, prospectively collected cohorts.

Nonetheless, this work advanced a conceptual framework in which tumour architecture was viewed as a functional determinant of the mechanical and immunological landscape. By demonstrating that NND could be systematically extracted from routine clinical specimens and correlated with molecular pathways and outcomes, we provided a scalable approach for integrating spatial biomechanics into cancer research. We proposed NND as a simple yet powerful digital pathology biomarker that captures a tumour's biomechanical state. The finding that mechanical stress and CD4^+^ T_Mem_ cells were spatially coupled opened new avenues for therapeutic intervention, suggesting that strategies aimed at modulating tumour mechanics could reshape the immune microenvironment and enhance immunotherapy efficacy. More broadly, this study revealed that the simple question of how close cells are to one another can illuminate previously unrecognized dimensions of tumour biology, addressing a fundamental gap in our understanding of how physical tissue organization shapes the tumour microenvironment and influences clinical outcomes.

## 4 METHODS AND MATERIALS

### 4.1 Publicly available H&E image datasets

All H&E‐stained whole slide images were obtained from public repositories hosted by TCIA.^1^ Below are the specific datasets used for each cancer type, with corresponding Digital Object Identifiers (DOIs), for each H&E slide, the tissue was assessed and recorded as either discontinuous fragments (DFs) or intact whole tissue: BRCA,^10^, ^11^ HER2‐BRCA,^12^ SLN‐BRCA,^13^ CCRCC,^14^ Non‐CCRCC,^14^ CM,^15^ COAD,^16^ CRC,^17^ DLBC,^18^ GBM,^19^, ^20^ Glioma,^21^ GEC,^22^ HGSOC,^23^ LCA,^24^ NLST,^25^ LSCC,^26^ LUAD,^27^ PCA,^28^ PDA,^29^ SAR,^30^ UCEC.^31^

### 4.2 Publicly available Bulk‐RNA datasets

The cancer genomics data used in this study were obtained from The Cancer Genome Atlas (TCGA) project. Multi‐omics data for the following 19 cancer types were downloaded from the official TCGA data portal, the Genomic Data Commons (GDC) Data Portal (https://portal.gdc.cancer.gov/)^43^: TCGA‐OV, TCGA‐READ, TCGA‐ESCA, TCGA‐HNSC, TCGA‐LUSC, TCGA‐TGCT, TCGA‐PAAD, TCGA‐STAD, TCGA‐KIRP, TCGA‐THCA, TCGA‐COAD, TCGA‐UCEC, TCGA‐PRAD, TCGA‐BLCA, TCGA‐LUAD, TCGA‐MESO, TCGA‐GBM, TCGA‐LIHC, TCGA‐PCPG, TCGA‐LGG, TCGA‐BRCA.

### 4.3 Publicly available single‐cell RNA sequencing datasets

We integrated five published single‐cell RNA‐seq datasets from the GEO database, encompassing over 300 000 cells across 34 cancer samples. After stringent filtering and quality control, the final dataset covered five distinct solid tumour types. The datasets were selected based on the following criteria: (1) Single‐cell RNA sequencing performed using the 10X Genomics platform; (2) Contained T‐cell data from cancer tissue; (3) Included detailed metadata on patient samples and clinical annotations; (4) Were from peer‐reviewed publications. The selected datasets represent melanoma (GSE123813),^44^ gastric cancer (GSE134520),^45^ cholangiocarcinoma (GSE138709),^36^ hepatocellular carcinoma (GSE149614)^37^, ^38^ and colorectal cancer (GSE178341).^39^

### 4.4 Publicly available spatial transcriptomics datasets

This study used spatially resolved transcriptomics to map mechanical signalling pathways in the human colon tumour microenvironment. We analysed five tissue samples profiled with the 10x Genomics Visium‐HD platform (10× Genomics, GSE280318),^40^ including two colon cancer samples (P1, P5) and two matched normal colon samples (P3, P5). Each sample contained a high density of spatially barcoded spots at subcellular resolution, enabling systematic profiling of interactions between specific immune and stromal compartments.

### 4.5 H&E image processing and niche analysis

#### 4.5.1 H&E image processing and nuclear segmentation

Digital slide acquisition: All datasets were downloaded in SVS (Aperio) format at native scanning resolutions (0.25–0.5 µm/pixel). Quality control procedures included the exclusion of whole‐slide images with suboptimal staining, tissue folding artefacts, or significant scanning defects. This screening process reduced the initial dataset of over 10 000 images to a final of 7910 images suitable for downstream computational analysis. Quality control procedures excluded whole‐slide images that failed to yield any detectable nuclei or were otherwise unprocessable by the segmentation pipeline.

### 4.6 Automated nuclear segmentation workflow for H&E‐stained images

All H&E‐stained whole slide images underwent automated nuclear segmentation using QuPath v0.4.0^41^ following a standardized following‐step pipeline:

### 4.7 Primary nuclei detection and annotation initialization

The initial segmentation was performed using watershed‐based cell detection on the Hematoxylin optical density (OD) channel. Parameters were optimized for H&E‐stained nuclei: pixel size of 0.25 µm, minimum nuclear area of 10 µm^2^, maximum area of 400 µm^2^, and a threshold of .05. Background subtraction was applied with an 8 µm radius.

### 4.8 Optional deep learning refinement

For challenging regions with high nuclear density or irregular morphologies, we employed an optional deep learning‐based segmentation using StarDist 2D with a pre‐trained model (dsb2018_heavy_augment.pb).^46^, ^47^ This method utilized the Hematoxylin OD channel at .5 µm/pixel resolution with percentile normalization (1%–99%) and a detection threshold of .5. The pre‐trained StarDist model (dsb2018_heavy_augment.pb)^46^, ^47^ segments all cell nuclei based on morphology but does not classify them by cell lineage (like tumour, immune, stromal). Consequently, all segmented nuclei were treated equally in NND calculation, and no cell‐type‐specific segmentation was performed prior to NND aggregation.

### 4.9 Quality control and validation

The segmentation pipeline incorporated multiple quality assurance measures: size filtering excluded nuclei outside the 10–400 µm^2^ range, watershed post‐processing ensured proper nuclear separation, and background subtraction eliminated non‐specific staining. Random manual verification of exported images confirmed segmentation accuracy, with visual inspection of nuclear boundaries and exclusion of artifacts.

This standardized workflow generated quantitative nuclear features including area (µm^2^), centroid coordinates, circularity, solidity, and Haematoxylin intensity, enabling comprehensive analysis of nuclear density distributions across all datasets.

### 4.10 Spatial analysis of cell nuclei aggregation

#### 4.10.1 Image processing and data acquisition

WSI of H&E‐stained tissue images were processed using automated software to identify and segment cell nuclei. Spatial coordinates (X, Y in ‘µm’) and classification data for each nucleus were extracted and saved in TSV format.

### 4.11 Calculation of spatial aggregation metrics

#### 4.11.1 Nearest Neighbour Distance (NND) computation

For each cell type within an image, the following computational steps were performed:

1. Coordinate matrix formation: X and Y coordinates of all nuclei belonging to the same cell type were organized into an *n* × 2 matrix, where n represents the number of nuclei.
2. Pairwise Euclidean distance calculation: All inter‐point distances were computed using the Euclidean distance formula:
$$d\_ij=\surd \left[{\left(x\_i\;-\;x\_j\right)}^{2}+{\left(y\_i\;-\;y\_j\right)}^{2}\right]$$
  where, $\left(x_{i},y_{i}\right)$ and $\left(x_{j},y_{j}\right)$ represent the coordinates of nuclei *i* and j, respectively.
3. Individual NND determination: For each nucleus *i*, its nearest neighbour distance was defined as the minimum distance to any other nucleus of the same type:
$$\mathrm{NN}D_{i}=\min_{j\neq i}\;d_{ij}$$
4. Distribution statistics: From the complete set of NND values $\left\{\mathrm{NN}D_{1},\mathrm{NN}D_{2},\text{…},\mathrm{NN}D_{n}\right\}$, the following statistics were derived: Mean NND: *NND_mean = (1/n) Σ NND_i*; Coefficient of variation: *CV_NND = σ_NND / NND_mean*.

### 4.12 Normalized NND for WSIs

1. Nuclear segmentation and fragment identification: For each H&E stained WSI, cell nuclei are segmented using a deep learning‐based pipeline (QuPath + StarDist). Each nucleus is assigned spatial coordinates (x, y). Tissue fragments (disconnected fragments, DFs) are identified based on spatial connectivity of nuclear coordinates.
2. Fragment level mean NND calculation: For a given fragment k containing n_k nuclei, the NND for each nucleus *i* is defined as the Euclidean distance to its nearest neighbouring nucleus within the same fragment. The fragment level mean NND is then calculated as the average of all NND_i in that fragment.
3. WSI level normalized NND calculation: For a WSI containing *K* fragments, the normalized NND is computed as the weighted mean of fragment level mean NNDs, with weights proportional to the number of nuclei in each fragment.
4. Handling of WSIs without fragmentation: For WSIs consisting of a single contiguous fragment, the formula simplifies to the fragment level mean NND.

Rationale for weighting: This weighting strategy ensures that larger, more biologically representative tissue fragments contribute proportionally more to the final WSI‐level metric, while small, potentially artefactual fragments, such as edge fragments with artificially large intercellular distances, have minimal influence.

### 4.13 Bulk‐RNA data processing

For each cancer type, we obtained gene expression data from CSV files containing survival‐associated transcriptomic profiles. We constructed gene expression matrices with genes, followed by rigorous quality control procedures to remove genes with zero expression across all samples. To ensure comparability across samples and platforms, we performed quantile normalization using the “preprocessCore” package,^48^ which standardizes the distribution of expression values. A total of 19 solid tumour types were retained for in‐depth downstream analysis.

### 4.14 Immune cell deconvolution methodology

Characterization of the tumour immune microenvironment was performed through computational deconvolution of bulk gene expression data. Our initial approach used the EPIC (Estimation of Proportions of Immune and Cancer cells) algorithm,^34^ which employs reference gene expression profiles to estimate the proportions of various cell types. However, due to compatibility issues encountered during implementation, we transitioned to the ‘immunedeconv’^32^ package, which provides a unified framework for multiple deconvolution methods.

The primary deconvolution method employed was ‘xCell’,^33^ which characterizes 64 distinct immune and stromal cell types using a signature‐based approach. ‘xCell’^33^ uses a curated set of gene signatures for each cell type and applies a spillover compensation technique to improve accuracy in complex tissue mixtures. For cancer types where ‘xCell’^33^ encountered computational challenges, we implemented MCPcounter^35^ (Microenvironment Cell Populations‐counter) as a fallback method. MCPcounter^35^ quantifies the abundance of eight immune and two stromal cell populations using carefully validated gene signatures.

The deconvolution process was applied independently to each cancer type's normalized expression matrix. Results were structured in a sample‐by‐cell‐type format, with each row representing an individual patient sample and columns containing estimated proportions for each cell type. Metadata including sample identifiers and cancer type designations were appended to facilitate subsequent integration and comparative analysis.

### 4.15 Cell type specification

The analysis focussed on immune and stromal cell populations including:

(1) Myeloid lineage: Macrophage (M1/M2), monocyte, neutrophil, myeloid dendritic cells (activated/resting), eosinophil, mast cell; (2) Lymphoid lineage: T‐cells (CD4^+^ memory/naive/central memory/effector memory/Th1/Th2/regulatory, CD8^+^ naive/central memory/effector memory, gamma delta), NK‐cells, B‐cells (naive/memory/plasma/class‐switched memory); (3) Progenitor cells: Common lymphoid progenitor, common myeloid progenitor, granulocyte‐monocyte progenitor, hematopoietic stem cell; (4) Stromal components: Endothelial cells, cancer‐associated fibroblasts; (5) Composite scores: Immune infiltration score (xCell), Stromal score (xCell).

### 4.16 Quality assurance for immune cell deconvolution

Comprehensive statistical summaries were generated for all cancer types and cell populations. For each combination, we calculated descriptive statistics including mean proportion, median proportion, standard deviation, quartiles and range. These metrics facilitated quantitative comparison of immune infiltration patterns and supported biological interpretation of observed differences.

We implemented several quality control measures throughout the analytical pipeline. Data type verification ensured that cell type proportion estimates were properly formatted as numerical values. Missing data were handled consistently across all analyses, with explicit documentation of sample sizes for each calculation. Methodological consistency was maintained through standardized function definitions and parameter settings.

The analysis pipeline incorporated robust error handling mechanisms, including fallback procedures for deconvolution methods and automated logging of processing outcomes. Computational efficiency was addressed through systematic memory management, including garbage collection between cancer type analyses to prevent resource exhaustion during large‐scale processing.

### 4.17 Composite microenvironment scores analysis

The composite microenvironment scores are calculated using established deconvolution algorithms from bulk RNA‐seq data. While the exact mathematical formulas are proprietary to the specific deconvolution tool used,^33^, ^34^, ^35^ three validated microenvironment scores were analysed alongside individual cell type proportions:

1. Immune score: Quantifies the overall level of immune cell infiltration in the tumour microenvironment. Calculation: Derived by aggregating the expression levels of immune‐specific genes (such as markers for T‐cells, B cells, NK cells, macrophages, dendritic cells). Typically, it is a weighted sum of immune cell gene signatures:
$$\mathrm{Immune}\;\mathrm{Score}\;=\sum_{i=1}^{n}w_{i}\;\cdot G_{i}$$
  where, $G_{i}$ are immune‐related genes and $w_{i}$ are their weights.
2. Stroma score: Measures the abundance of stromal components (such as fibroblasts, endothelial cells, extracellular matrix). Calculation: Based on stromal‐specific gene signatures collagen genes, fibroblast markers, endothelial markers). Similar to the immune score, it is a weighted sum:
$$\mathrm{Stroma}\;\mathrm{Score}\;=\sum_{j=1}^{m}w_{j}\;\cdot S_{j}$$

where, $S_{j}$ are stromal‐related genes.

### 4.18 Data integration framework

The study integrated two complementary datasets: (1) histopathological image‐derived spatial metrics (cellular density and nearest neighbour distance), and (2) transcriptomics‐based cell proportion estimates from TCGA pan‐cancer cohort.

### 4.19 Cancer type harmonization

Cancer type designations were standardized between datasets using a bidirectional mapping system, allowing cross‐dataset comparison of matched cancer types. Data were aggregated to cancer‐type level by calculating median values for spatial metrics and cell proportions. To enable cross‐dataset integration, cancer type designations were harmonized using a bidirectional mapping system as TCGA nomenclature (e.g., ‘TCGA‐COAD’, ‘“TCGA‐LUAD”’) to the abbreviated clinical designations (e.g., ‘COAD’, ‘LUAD’).

### 4.20 Single‐cell RNA sequencing analysis

*Data preprocessing and quality control*: Raw gene expression matrices for each dataset were processed independently using the Seurat package (v4) in R. The preprocessing steps included:

(1) Initial Dataset Sizes: The datasets contained the following total cell counts before T‐cell subtyping: GSE123813 (53 780 cells), GSE134520 (32 332 cells), GSE138709 (31 302 cells), GSE149614 (> 70 000 cells), and GSE178341 (371 223 cells); (2) Quality Control: For each dataset independently, cells with unique feature counts (genes) less than 200 or greater than 6000, and with mitochondrial gene content exceeding 15% were filtered out to remove low‐quality cells or potential doublets; (3) Normalization and Scaling: Gene expression counts were normalized using the ‘LogNormalize’ method with a scale factor of 10 000. Highly variable features (2000 genes) were identified using the ‘vst’ method in Seurat^42^; (4) Batch Effect Correction (within datasets): For datasets containing multiple samples or patients, potential batch effects were mitigated using the ‘IntegrateData’ function in Seurat^42^ (anchor‐based integration) to enable joint downstream analysis.

### 4.21 Data normalization and integration

Expression matrices were normalized using the ‘SCTransform’ method^49^ with regularization to remove technical variation while preserving biological heterogeneity. Multiple datasets were integrated using reciprocal principal component analysis (RPCA) to correct for batch effects while maintaining biological variation across cancer types.

### 4.22 T‐Cell identification

Given the transcriptional similarity among T‐cell subsets and the integration of multiple cancer types, T‐cell subtypes were defined based on canonical marker gene expression at the single‐cell level rather than relying solely on cluster identity, a strategy commonly used in multi‐dataset T‐cell atlas studies.^50^, ^51^, ^52^ After annotation, subtype identities were mapped back to the clustering results to examine their distribution across clusters.

T‐cells were identified from the integrated object for each dataset using canonical marker genes:

(1) Pan‐T cell markers: CD3D, CD3E, CD3G; (2) Subset Confirmation: CD4 (Helper T‐cells), CD8A (Cytotoxic T‐cells). Cells positively expressing these markers were subset for all subsequent cancer‐type‐specific downstream analyses. The T‐cell identification resulted in the following cell counts per dataset: (1) GSE123813 (melanoma): 53 780 T‐cells (from original 53 780 total cells); (2) GSE134520 (gastric cancer): 255 T‐cells (from original 32 332 total cells); (3) GSE138709 (cholangiocarcinoma): 299 T‐cells (from original 31 302 total cells); (4) GSE149614 (hepatocellular carcinoma): 2534 T‐cells (from original > 70 000 total cells); (5) GSE178341 (colorectal cancer): 9,295 T‐cells (from original 371 223 total cells). Total T‐cells analysed: 66 163 high‐quality T‐cells across 5 datasets after quality control and for subtyping.

### 4.23 Integrated analysis of All T‐Cells

Importantly, unsupervised clustering was performed to group cells based on their microenvironmental similarity and shared ecological niches rather than lineage identity alone.

For comprehensive T‐cell characterization, we integrated all filtered T‐cells from the five datasets and performed the following analyses: (1) Integrated Clustering: All 66 163 T‐cells were integrated using the Harmony algorithm to correct for technical batch effects while preserving biological variance. The integrated data was scaled, and principal component analysis (PCA) was performed on highly variable genes; (2) UMAP Generation: The top 30 principal components (determined by elbow plot and JackStraw analysis) were used for Uniform Manifold Approximation and Projection (UMAP) dimensionality reduction. The UMAP embedding was computed with the following parameters: n.neighbours = 30, min.dist = 0.3, metric = ‘cosine’. A 21‐cluster structure (clusters 0–20) was either identified from existing metadata or generated de novo using k‐means clustering on UMAP coordinates (k = 21, nstart = 25, iter.max = 100). UMAP visualizations were created to display T‐cell subtype distributions and cluster assignments. This resulted in the UMAP visualization showing all T‐cells in a two‐dimensional space.

### 4.24 T‐Cell subset classification and annotation

Single‐cell RNA sequencing data from five public datasets (GSE123813, GSE134520, GSE138709, GSE149614, GSE178341) were integrated and analysed using the Seurat pipeline. Unsupervised clustering analysis identified 21 distinct T‐cell clusters, which were visualized using Uniform Manifold Approximation and Projection (UMAP). T‐cell subsets were annotated based on canonical marker gene expression and previously established transcriptional signatures, and further divided into the following detailed subtypes:

1. Activated T‐cells were identified by high expression of activation markers including CD69, HLA‐DRA, IL2RA (CD25) and TNFRSF4 (OX40), indicating recent or ongoing T‐cell receptor stimulation.
2. CD4+ T‐cell subsets were defined by CD4, CD3D, CD3E, and CD3G expression, and further stratified into functional and differentiation states. CD4_naive cells were characterized by CCR7, SELL (CD62L), TCF7, and LEF1 expression, representing antigen‐inexperienced CD4+ T‐cells. CD4_central_memory cells were defined by CCR7, SELL, IL7R (CD127) and TCF7 expression, with capacity for lymph node homing and rapid proliferation upon re‐stimulation. CD4_effector_memory cells lacked CCR7 and SELL expression while expressing IL7R, GZMK, and KLRG1, enabling rapid effector function in peripheral tissues. CD4_resident_memory cells were identified by ITGAE (CD103), ITGA1 (CD49a), CXCR6, and RORA expression, representing tissue‐resident populations. CD4_Tfh (follicular helper T‐cells) were marked by BCL6, CXCR5, PDCD1, and ICOS expression, specialized for providing B cell help in germinal centres. CD4_Th1 cells were defined by TBX21 (T‐bet), IFNG, CXCR3, and STAT1 expression, promoting cell‐mediated immunity. CD4_Th2 cells were characterized by GATA3, IL4, IL5, IL13, and CCR4 expression, driving humoral and allergic responses. CD4_Th17 cells were identified by RORC (RORγt), IL17A, IL17F, CCR6, and IL23R expression, mediating neutrophil recruitment and mucosal defence. CD4_Treg (regulatory T‐cells) were marked by FOXP3, IL2RA (CD25), CTLA4, and IKZF2 (Helios) expression, responsible for immune suppression and homeostasis.
3. CD8+ T‐cell subsets were identified by CD8A and CD8B expression and divided into differentiation states. CD8_naive cells expressed CCR7, SELL, TCF7, and LEF1. CD8_central_memory cells were defined by CCR7, SELL, IL7R, and EOMES expression. CD8_effector_memory cells lacked CCR7 and SELL, with expression of GZMK, GZMB, PRF1, and KLRG1. CD8_terminally_differentiated cells were characterized by high expression of cytotoxic molecules including GZMB, PRF1, GNLY, and NKG7, along with KLRG1 expression and loss of memory‐associated genes such as IL7R, representing end‐stage effector cells with limited proliferative capacity.
4. Cytotoxic T‐cells were identified based on high expression of cytotoxic effector molecules including GZMA, GZMB, GZMK, PRF1, GNLY, and NKG7, along with the transcription factors EOMES and TBX21. This population predominantly comprised activated and terminally differentiated CD8+ T‐cells.
5. Exhausted T‐cells were defined by sustained co‐expression of multiple inhibitory receptors including PDCD1, LAG3, HAVCR2, TIGIT, and CTLA4, along with exhaustion‐associated transcription factors TOX, TOX2, and NR4A1, accompanied by reduced effector cytokine expression.
6. MAIT‐cells (mucosal‐associated invariant T‐cells) were characterized by SLC4A10, TRAV1‐2, KLRB1 (CD161), and IL18R1 expression, representing innate‐like T‐cells recognizing bacterial vitamin B metabolites.
7. Migrating T‐cells were identified by high expression of chemokine receptors and adhesion molecules involved in trafficking, including CXCR3, CCR5, CCR2, ITGB1 (CD29), and ITGAL (CD11a), indicating active migratory potential.
8. T_cell_general comprised a baseline population expressing core T‐cell lineage genes including CD3D, CD3E, CD3G, and TRAC without strong enrichment for specific functional or differentiation markers, representing a mixed or transitional state.
9. Low_confidence_T‐cells included cells with ambiguous transcriptional signatures, low RNA counts, or partial marker expression that precluded definitive subset assignment. These cells were retained for analysis but interpreted with caution.

All subset annotations were validated by differential gene expression analysis and reference to established T‐cell transcriptional signatures.

### 4.25 Identification of CD4^+^ Memory T‐Cells

CD4^+^ memory T‐cells were identified using a dual‐marker approach. CD4^+^ T‐cells were defined by expression of CD4, CD3D and CD3E markers, while memory phenotype was determined by expression of memory‐associated markers (CCR7, SELL, CD44, IL7R). Cells were classified as CD4^+^ memory T‐cells if they met the following criteria: (1) Expressed > 50% of available CD4 markers; (2) Expressed > 30% of available memory markers, which the manual curation of marker expression patterns and comparison with established memory T‐cell signatures; (3) Showed minimal expression of CD8 markers (< 30% of CD8A/CD8B markers). CD4+ naive T cells and CD4+ central memory T cells share expression of CCR7, SELL, and TCF7. They were distinguished based on IL7R (CD127) expression: naive cells were defined as IL7R‐low/negative, while central memory cells were defined as IL7R‐high. This distinction is critical because CCR7/SELL/TCF7 alone cannot separate these two populations. CD4_effector_memory cells lacked CCR7 and SELL expression while expressing IL7R, GZMK, and KLRG1. CD4_resident_memory cells were identified by ITGAE, ITGA1, CXCR6, and RORA expression.

### 4.26 Comprehensive T‐Cell subtype annotation

A systematic annotation of T‐cell subtypes was performed using predefined gene signatures for 14 distinct T‐cell populations, including: (1) CD4^+^ subsets: Th1, Th2, Th17, Tfh, Treg, naïve, central memory, effector memory and resident memory; (2) CD8+ subsets: naïve, central memory, effector memory and terminally differentiated; (3) Other T‐cells: γδ T‐cells, NKT‐cells, and MAIT‐cells.

### 4.27 Pseudo‐time analysis of CD4^+^ memory T‐Cell differentiation trajectories

We reconstructed developmental trajectories of CD4^+^ memory T‐cells using Slingshot pseudo‐time analysis. Cluster 0, characterized by naïve markers (CCR7, SELL, TCF7, LEF1), was designated as the starting point (root). This cluster consists of naïve CD4^+^ T cells, which are not classified as memory cells but represent the differentiation origin for memory subsets. Cells were ordered along differentiation trajectories based on transcriptional similarity, with pseudo‐time values ranging from 0 (least differentiated) to 1 (most differentiated). Genes significantly associated with pseudo‐time progression were identified using Spearman correlation (|*ρ*| > .3, FDR < .05). Within cluster 2, we separately analysed colorectal cancer and melanoma cells to compare differentiation patterns between cancer types.

### 4.28 Differential expression analysis for CD4^+^ memory T‐Cell

Memory T‐cells were identified from an integrated single‐cell RNA sequencing dataset containing 15 914 CD4^+^ T‐cells using three complementary approaches: (1) gene signature scoring based on the Memory_CD4 signature score, (2) cell type annotations in the metadata, and (3) expression validation of canonical memory markers (CCR7, SELL, IL7R, CD27, CD28, CD45‐RO). Cells meeting these criteria formed the memory T‐cell subset for subsequent analysis.

Differential gene expression analysis was performed to compare memory T‐cells localized within cluster 2 versus memory T‐cells distributed across all other clusters (0–20). Statistical analysis was conducted using a Wilcoxon rank‐sum test with a custom pipeline designed for Seurat v5's layered data structure. Analysis parameters included a minimum log2 fold change threshold of .25, a minimum expression threshold of 5% of cells in either group, and Benjamini‐Hochberg false discovery rate adjustment with a significance threshold of adjusted *p*‐value < .05. Gene set enrichment analysis was performed on significantly differentially expressed genes to identify relevant biological pathways and functions.

### 4.29 Computational analysis of spatial transcriptomics data

#### 4.29.1 Single‐cell RNA sequencing data processing

Spatial transcriptomics data from four Visium HD human colon samples (two cancer: Cancer_P1 and Cancer_P5; two adjacent normal: Normal_P3 and Normal_P5) were analysed using Seurat (v4.0+). Each sample was loaded from previously processed Seurat objects containing dimensional reduction and spatial coordinate information. Spatial coordinates (*x, y*) were extracted for downstream visualization when available. For spatial transcriptomics analysis (Figure 5), the same mechanical gene signature (composite Mechanical Score) was used for two purposes: (1) to stratify CD4^+^ memory T cells into high versus low mechanical stress groups using the median score within each tissue section as the cutoff, and (2) to compare pathway activity between the resulting groups. Thus, this analysis demonstrates a coherent expression gradient along the defined mechanical axis rather than providing independent validation.

### 4.30 T‐Cell subtype classification

T‐cells were identified based on expression of CD3 complex genes (CD3D, CD3E, CD3G). Cells with expression scores above the 60th percentile were classified as T‐cells. Subsequently, T‐cells were assigned to functional subtypes using a marker‐based scoring approach. Eleven T‐cell subtypes were defined using established lineage‐specific markers:

(1) Naive CD4^+^ T‐cells: CCR7, SELL, TCF7, LEF1, CD4; (2) Memory CD4^+^ T‐cells: IL7R, CD4; (3) Th1 cells: IFNG, TBX21, CD4; (4) Th2 cells: GATA3, IL4, CD4; (5) Th17 cells: RORC, IL17A, CD4; (6) Regulatory T‐cells (Treg): FOXP3, IL2RA, CTLA4, CD4; (7) Naive CD8^+^ T‐cells: CCR7, SELL, CD8A, CD8B; (8) Effector CD8^+^ T‐cells: GZMB, PRF1, IFNG, NKG7, CD8A, CD8B; (9) Exhausted CD8^+^ T‐cells: PDCD1, HAVCR2, LAG3, TIGIT, CD8A, CD8B; (10) Cytotoxic T‐cells (pan‐subtype): GZMA, GZMB, PRF1, NKG7; (11) Exhaustion signature (pan‐subtype): PDCD1, CTLA4, LAG3, TIGIT, HAVCR2.

For each cell, subtype scores were calculated as the mean expression of available markers for each category. Cells were assigned the subtype with the highest score exceeding a threshold of .2; cells not meeting this criterion were designated as ‘Unassigned’.

### 4.31 Mechanical signalling pathway scoring

Five mechanical signalling pathway modules were defined based on literature‐curated gene sets:

(1) Core Hippo pathway: MST1, MST2, LATS1, LATS2, YAP1, WWTR1 (TAZ), TEAD1, TEAD4; (2) Hippo regulators: NF2, AMOT, AMOTL1, AMOTL2; (3) YAP/TAZ transcriptional targets: CTGF, CYR61, ANKRD1, MYC, CCND1; (4) Mechanical sensors: ITGA1, ITGA2, ITGB1, ITGB3, PTK2 (FAK), VCL, RHOA, ROCK1; (5) Mechanotransduction components: PIEZO1, PIEZO2, LMNA, LMNB1; (6) T‐cell mechanical function genes: CD3D, CD3E, CD247, ZAP70, LAT, CD28, ICAM1, SELL, CXCR4, CCR7, CD44.

For each cell, pathway scores were calculated as the mean expression of all available genes within each module. Two composite scores were derived:

(1) Hippo Score: Mean of Core Hippo, Hippo regulators, and YAP/TAZ target modules; (2) Mechanical Score: Mean of Mechanical sensors, Mechanotransduction and T‐cell mechanical modules

### 4.32 Pathway score calculation

For each CD4 memory T‐cell, pathway activity scores were computed as:

$$\begin{array}{ccc} & & Pathway\;Score=\\ & & \;\left(1/n\right)\;*\;\Sigma \left(log-normalized\;expression\left(gene\_i\right)\right) \end{array}$$

where: n = number of available pathway genes, Σ = sum from i = 1 to n, gene_i = individual gene in the pathway.

For spatial transcriptomics analysis (Figure 5), the same mechanical gene signature (composite Mechanical Score) was used for two purposes: (1) to stratify CD4^+^ memory T cells into high versus low mechanical stress groups using the median score within each tissue section as the cutoff, and (2) to compare pathway activity between the resulting groups. Thus, this analysis demonstrates a coherent expression gradient along the defined mechanical axis rather than providing independent validation

### 4.33 Spatial analysis of mechanical signalling

Spatial coordinates (*x, y* positions) from the Visium‐HD platform were used to map pathway activity across tissue sections. Pathway scores were visualized as colour gradients on tissue maps, enabling identification of spatial patterns and microenvironmental correlations.

### 4.34 Survival analysis of hippo mechanical signalling and CD4^+^ memory T‐Cell interactions

#### 4.34.1 Patient cohort and data acquisition

Clinical and molecular data were obtained from The Cancer Genome Atlas (TCGA) pan‐cancer cohort for the 19 solid tumours with complete clinical follow‐up information including: (1) Overall survival (OS) was defined as the time from diagnosis to death from any cause; (2) Patients alive at last follow‐up were censored; (3) Survival time (days) and vital status (dead/alive) were extracted from clinical records.

### 4.35 Gene selection

Two gene sets were selected for analysis:

1. Mechanical Signalling Genes (Hippo Pathway): Core Hippo pathway components: YAP1, TAZ (WWTR1), TEAD1‐4; Upstream regulators: LATS1‐2, MST1‐2, SAV1, MOB1A‐B; Downstream targets: CTGF, CYR61; Angiomotin family: AMOT, AMOTL1‐2, ANKD1.
2. CD4^+^ Memory T‐Cell Signature Genes: T‐cell markers: CD4, IL7R (CD127); Memory/naive markers: CCR7 (CD197), SELL (CD62L); Transcription factors: LEF1, TCF7.
3. Gene expression normalization: Raw expression values were log2‐transformed for normalization**;** Missing values were imputed using *k*‐nearest neighbours algorithm (*k* = 10); Expression values were standardized within each cancer type to account for batch effects.

### 4.36 Scoring methodology

#### 4.36.1 Composite score calculation

For each patient, two composite scores were calculated:

(1) Mechanical Signalling Score (MSS): MSS = mean (expression of all 20 mechanical signalling genes); (2) CD4^+^ Memory T‐Cell Score (CD4MS): CD4MS = mean (expression of all 6 CD4^+^ memory T‐cell signature genes).

### 4.37 Stratification by median expression

For each cancer type separately: (1) Patients were classified as ‘high’ if their score exceeded the median value; (2) Patients were classified as ‘low’ if their score was at or below the median value. (3) Inclusion Criteria: For each cancer type analysis: Minimum sample size: 20 patients; Minimum events (deaths): 5 per group; Each comparison group required ≥5 patients. We performed seven distinct survival analyses:

For dual‐factor survival analyses (Figure 6C–E), patients within each cancer type were stratified into four subgroups based on median cutoffs for both Mechanical Signalling Score (MSS) and CD4^+^ Memory T cell Score (CD4MS): Double High (MSS high / CD4MS high), Double Low (MSS low / CD4MS low), Mechanical High / CD4 Low (MSS high / CD4MS low), and Mechanical Low / CD4 High (MSS low / CD4MS high). For Kaplan–Meier comparisons, each specific subgroup was compared against all other patients combined as the remaining three subgroups. This approach prioritizes statistical power but has the limitation that the reference group is heterogeneous in biological composition.

### 4.38 Statistical analysis

One‐sample *t*‐tests: Each cancer type's mean density compared to the global mean across all 21 cancer types. Benjamini‐Hochberg false discovery rate (FDR) with *α* = .05. To address the issue of multiple comparisons, we applied the Benjamini–Hochberg FDR correction to the raw *p*‐values. Significance levels were defined as: FDR‐adjusted *p* < .05*; FDR‐adjusted *p* < .01**; FDR‐adjusted *p* < .001***.

For heatmap visualization (Figure 3C,D,F), Spearman rank correlations were calculated between spatial metrics (overall cell density and normalized nearest neighbour distance, NND) and each of the deconvoluted immune cell type fractions and signalling pathway scores across the nine histologically matched cancer types. For heatmap visualization, only the top‐ranked associations based on absolute Spearman's *ρ* values were displayed. Specifically, for Figure 3E (cell density), the top associations with the highest |*ρ*| values were shown; for Figure 3F (NND), the top associations with the highest |*ρ*| values were shown. Associations with very small |*ρ*| values (indicating weak or negligible correlations) were omitted from the main figures to direct attention to the most prominent patterns. Statistical significance for the displayed associations was indicated by stars: *p < 0.05, **p < 0.01, *p < .001. Non‐significant associations (*p* ≥ .05) are displayed without stars. Full correlation matrices, including all cell types, pathways, *ρ* values, and *p*‐values, are provided in Table S3.

Spearman rank correlations were computed between spatial metrics and cell type proportions at the cancer‐type level. Multiple testing correction was applied using Benjamini–Hochberg FDR method. Individual scatter plots were generated with top‐left statistical analysis. Significance thresholds were: *** (*p* < .001), ** (*p* < .01), * (*p* < .05). Kaplan–Meier Survival Analysis: Survival curves were estimated using the Kaplan–Meier method, Log‐rank test was used to compare survival distributions between groups, *p*‐values < .05 were considered statistically significant.

### 4.39 Softwares

Image analysis was conducted using QuPath (v0.6.0). Subsequent statistical analyses and visualizations were performed in R (v4.4.3). The following R packages were employed for general data processing and visualization: readr and dplyr for data handling; ggplot2, ggrepel, umap, ComplexHeatmap, Hmisc and PerformanceAnalytics for visualization, dimensionality reduction, and statistical plotting.

For single‐cell RNA sequencing (scRNA‐seq) data analysis, we primarily utilized Seurat (v4) for cell clustering, visualization and differential expression analysis, and clusterProfiler for functional enrichment analysis.

For spatial transcriptomics data analysis, the following packages and versions were used: Seurat (v4.3.0) for data integration, normalization, and spatial visualization; ggplot2 (v3.4.2) for generating figures; ComplexHeatmap (v2.14.0) for pathway activity heatmaps; circlize (v0.4.15) for circular visualizations of gene‐pathway networks; viridis (v0.6.2) and RColorBrewer (v1.1.3) for color palettes; patchwork (v1.1.2) for arranging multi‐panel plots; and dplyr (v1.1.2) with tidyr (v1.3.0) for data wrangling.

For survival analysis, survival (v3.5‐5) for survival analysis, nd survminer (v0.4.9) for visualization.

## AUTHOR CONTRIBUTIONS

**Linglong Huang**: Conceptualization, methodology, software, formal analysis, data curation, investigation, visualization, writing of the original draft, writing, review and editing, project administration, funding acquisition. **Arne Östman**: Supervision, writing, review and editing, resource, funding acquisition. **Yunfan Sun**: Supervision, writing, review and editing, resources. All authors read and approved the final manuscript.

## CONFLICT OF INTEREST STATEMENT

The authors declare no conflicts of interest.

## CONSENT FOR PUBLICATION

All authors have reviewed and approved the final version of this manuscript and consent to its submission and publication in *Biomarker Research*. This manuscript has not been published previously and is not under consideration elsewhere.

## ETHICS STATEMENT

All data used in this study are publicly available from The Cancer Genome Atlas (TCGA), The Cancer Imaging Archive (TCIA), and the Gene Expression Omnibus (GEO) repositories. The datasets were collected under institutional review board‐approved protocols with patient consent obtained by the original contributors. As this study involved secondary analysis of publicly available and anonymized data, no additional ethics approval was required. All methods were performed in accordance with the relevant guidelines and regulations.

## Supporting information



Supporting Information

Supporting Information

Supporting Information

Supporting Information

Supporting Information

Supporting Information

Supporting Information

Supporting Information

Supporting Information

## Data Availability

The datasets supporting the findings of this study are available from public repositories as described in the Methods section. H&E‐stained whole‐slide images are accessible via The Cancer Imaging Archive (TCIA) with DOIs listed in Methods. Bulk RNA‐seq data are available through The Cancer Genome Atlas (TCGA) via the Genomic Data Commons (GDC) Data Portal (https://portal.gdc.cancer.gov/). Single‐cell RNA‐seq datasets are accessible through the Gene Expression Omnibus (GEO) under accession numbers GSE123813, GSE134520, GSE138709, GSE149614 and GSE178341. Spatial transcriptomics data are available under GSE280318. The datasets supporting the findings of this study are available from public repositories as described in the Methods section. Processed data, including nuclear segmentation outputs, spatial metrics, and deconvolution results, are available from the corresponding author (Linglong Huang, linglong.huang@ki.se) upon reasonable request.
