# Supplementary material for: Pan‐cancer atlas of cellular architecture reveals a nearest‐neighbour distance‐associated biomechanical‐immune axis involving CD4+ memory T cells
Source: Clin Transl Med. 2026 Jul 21;16(7):e70742. doi: 10.1002/ctm2.70742 (PMC13388988; doi:10.1002/ctm2.70742)
Supplement: Supplementary file 1 — Supporting Information [file CTM2-16-e70742-s001.docx]

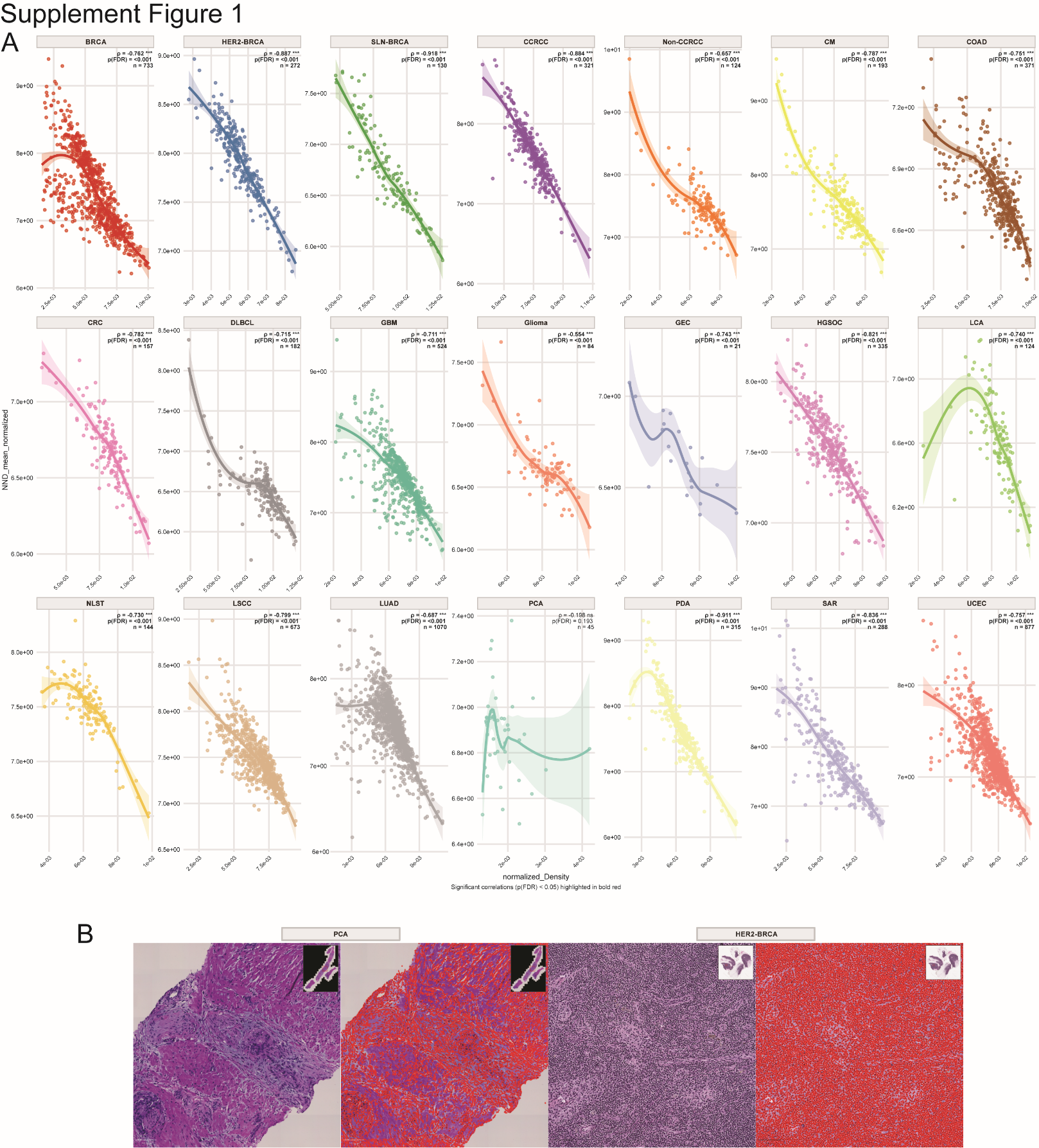


**Supplement Figure 1: The Correlation between Normalized NND and Cell Density.** A: Scatter plots showed the Spearman correlation between NND and overall cell density was analyzed across 21 TCGA solid tumor types, with p-values corrected for multiple comparisons using the FDR method (Exclude WSIs that consist entirely of zero values, or have an NND-to-density ratio exceeding 5000.) B: HE-based tissue segmentation comparing cellular distribution patterns between PCA (low NND, low density) and SLN-BRCA (low NND, high density). Scale bar: 100µm. Significance levels were set at *p < 0.05, **p < 0.01, and ***p < 0.001 (FDR-adjusted). Non-significant results (p > 0.05) are not denoted. Details were available in Supplement Table S1. Abbreviations: NND, Nearest Neighbor Distance; FDR, False Discovery Rate.

**
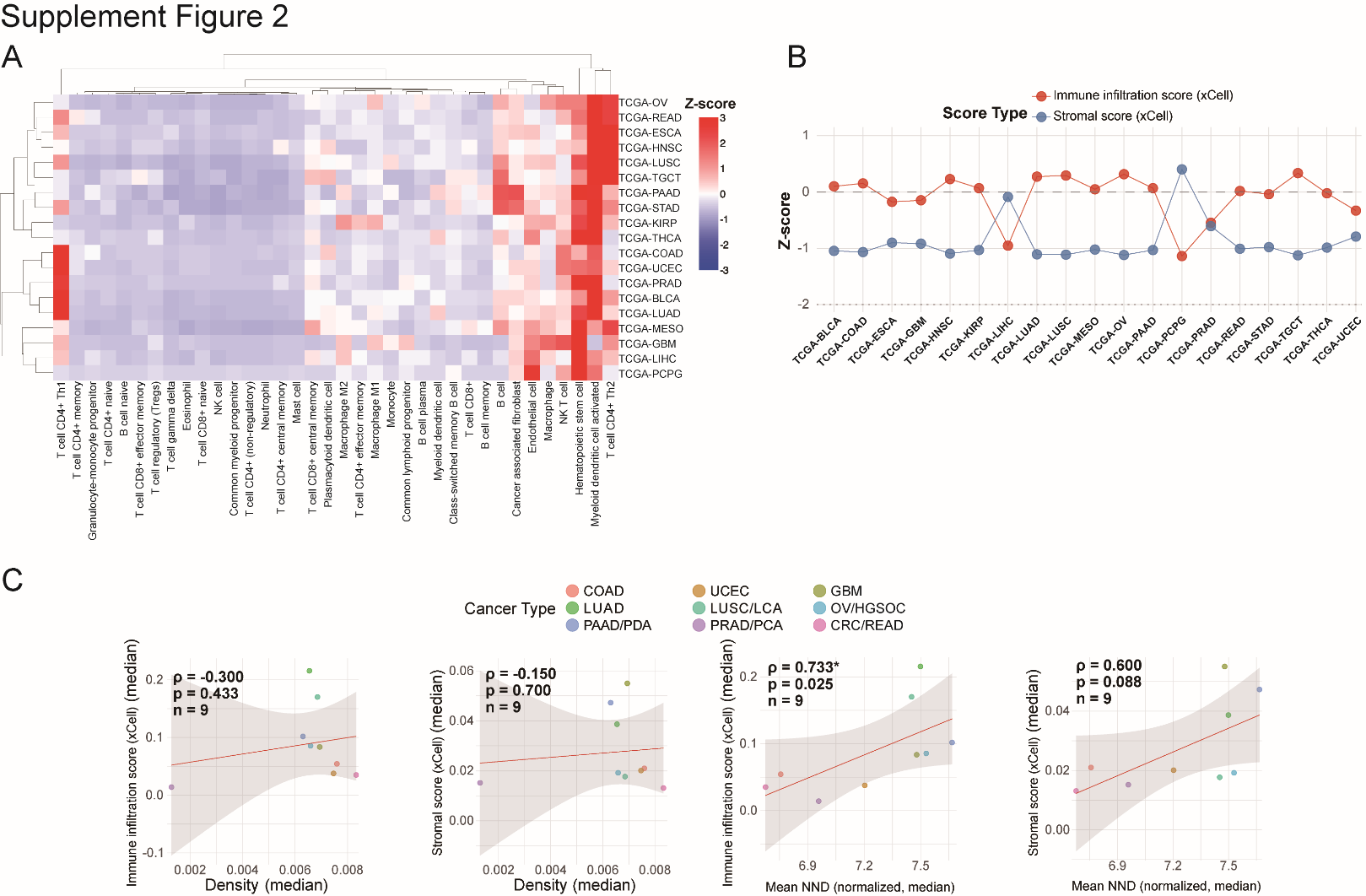
**

**Supplement Figure 2: Deconvolution of T-cell Subtypes Across TCGA Solid Tumors.** A: Unsupervised clustering and heatmap visualization of z-score normalized cell type fractions across solid tumors, as estimated by deconvolution from bulk RNA‑seq data. B: Line plot of immune and stroma metrics. Showing immune and stroma scores across solid tumors. Details were listed in the Method section. C: A scatter plot analysis was performed to investigate the relationship between Immune infiltration score (xCell) and stromal score (xCell) and the spatial organization of nine different cancer types. Specifically, we assessed how these scores correlate with overall cell density and the normalized NND. Associations were assessed using Spearman’s rank correlation. Significance levels are denoted as follows: *p < 0.05, **p < 0.01, and ***p < 0.001 (all p-values are FDR-adjusted). Non-significant correlations (p > 0.05) are not marked. Abbreviations: NND, Nearest Neighbor Distance; FDR, False Discovery Rate.


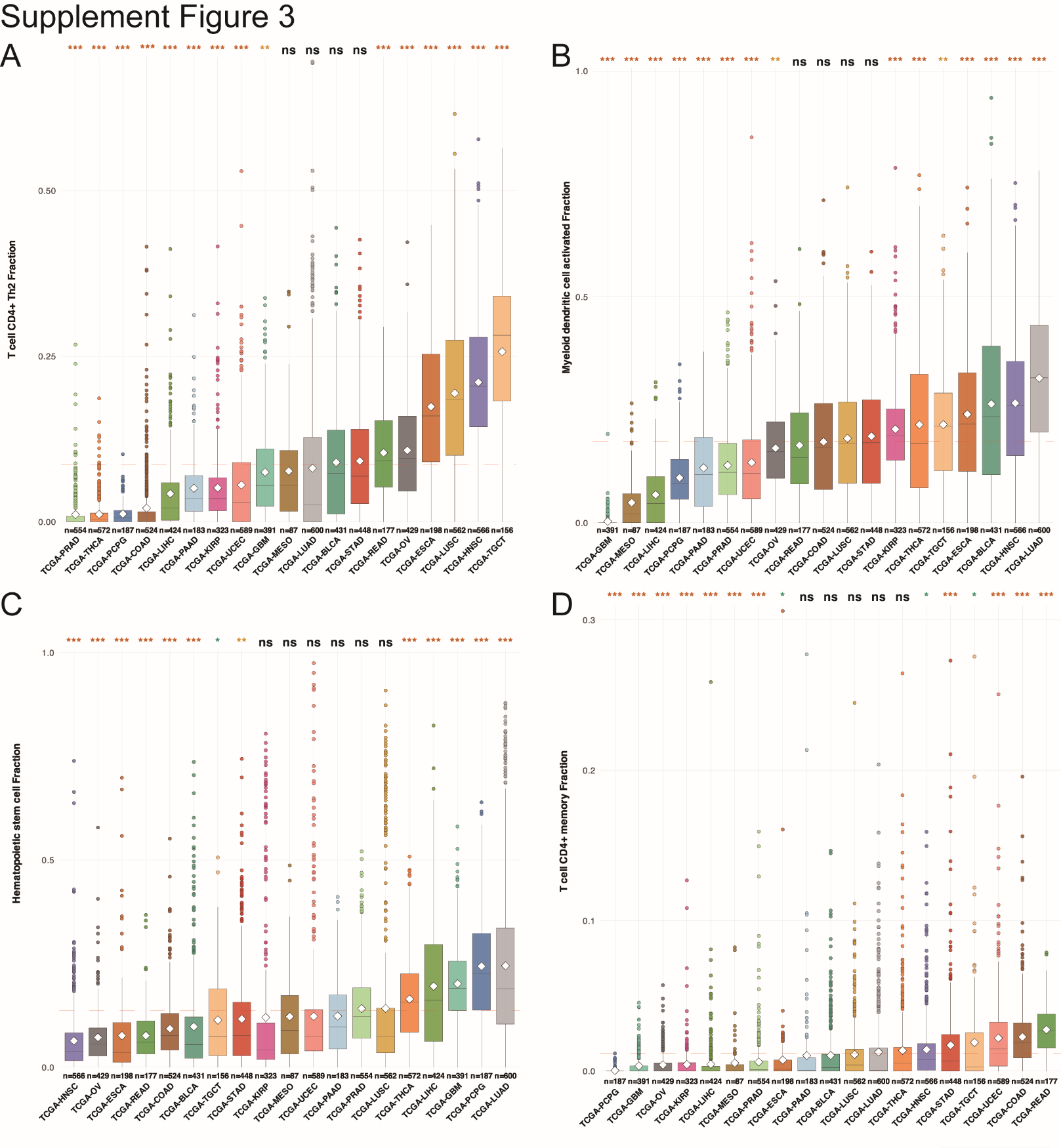


**Supplement Figure 3: Distribution of Four Selected Immune Cell Types Across TCGA Solid Tumors.** A-D: Boxplots show the distribution of subset fractions for: (A) CD4⁺ Th2 cells, (B) Myeloid dendritic cells, (C) Hematopoietic stem cells, and (D) CD4^+^T_Mem_ cells. The red horizontal line in each plot indicates the overall mean across all tumors. Statistical significance of deviations from the overall mean was assessed using one-sample tests, with p-values corrected for multiple comparisons by the FDR method. Statistical significance was defined at three levels: *p < 0.05, **p < 0.01, and ***p < 0.001 (all reported p-values are FDR-adjusted). Non-significant associations (p > 0.05) are not annotated. Non-significant results (p > 0.05) are not denoted. Abbreviation: CD4⁺ memory T-cells, CD4^+^T_Mem_ cell; NND, Nearest Neighbor Distance; FDR, False Discovery Rate.


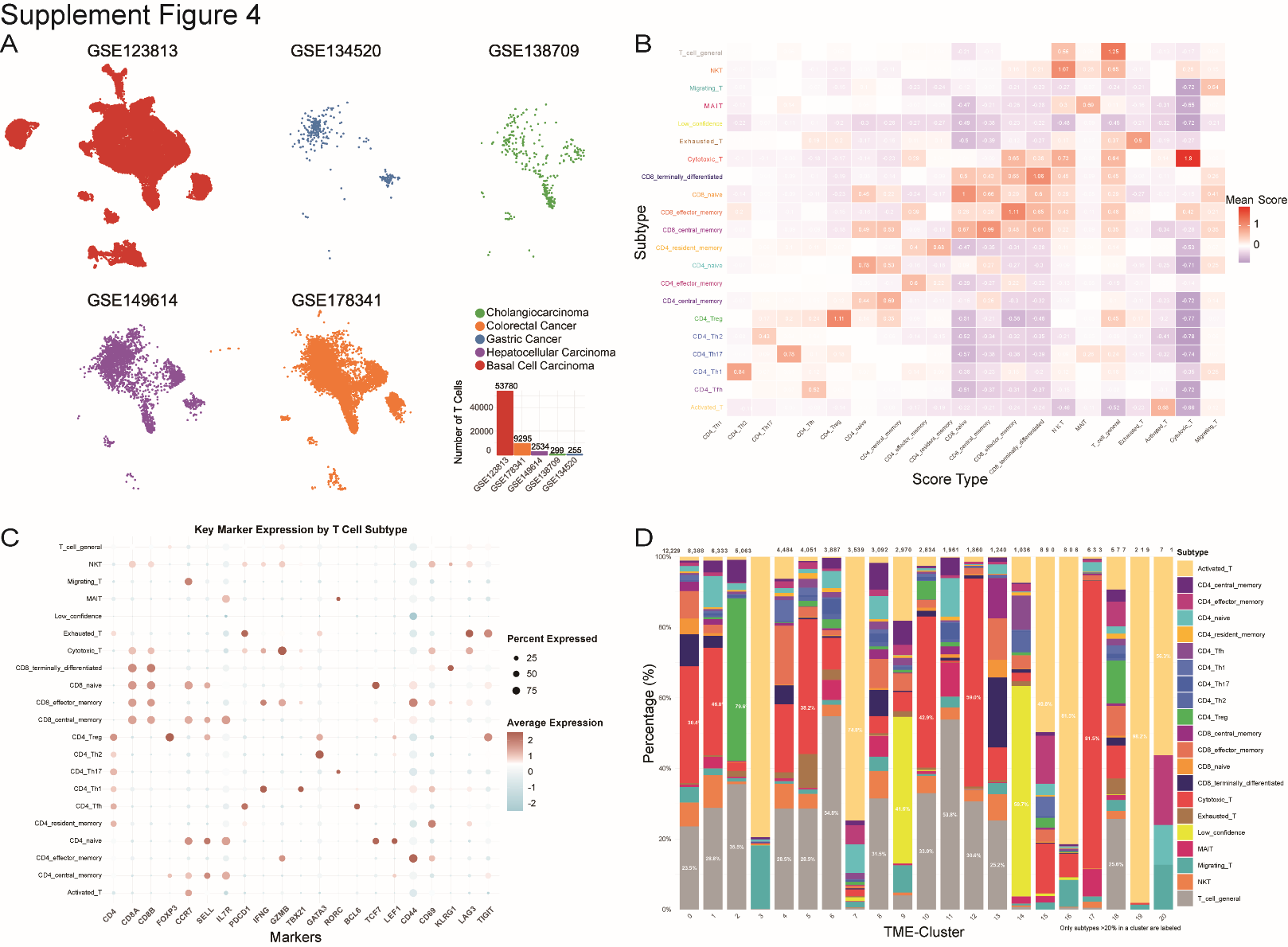


**Supplement Figure 4: Distribution of T-Cell Subtypes Across 21 UMAP Clusters.** A: UMAP visualization and cellular composition of integrated datasets. UMAP plot (left) showing the overall distribution of all samples. Bar plot (bottom right) quantifying the number of T-cells per individual dataset. B: Heatmap depicting the distribution of T cell subtype scores and corresponding subtype annotations. The full list of subtypes we examined included: T_cell_general, NKT, Migrating_T, Low_confidence, Exhausted_T, Cytotoxic_T, CD8_terminally_differentiated, CD8_naive, CD8_effector_memory, CD8_central_memory, CD4_Treg, CD4_Th2, CD4_Th17, CD4_Th1, CD4_resident_memory, CD4_naive, CD4_effector_memory, CD4_central_memory, and Activated_T. C: Dot plot of key marker expression across T cell subtypes. Size of each dot represents the percentage of cells expressing the marker, and the color represents the average expression level. D: The bar plots showed the percentage distribution of T cell subsets across the 21 identified subtypes. Subtypes that accounted for more than 20% of a cluster were specifically labelled.
